# Using Gjd3-CreEGFP mice to examine atrioventricular node morphology and composition

**DOI:** 10.1038/s41598-019-38683-8

**Published:** 2019-02-14

**Authors:** Samadrita Bhattacharyya, Jialei Duan, Lin Wang, Boxun Li, Minoti Bhakta, Antonio Fernandez-Perez, Gary C. Hon, Nikhil V. Munshi

**Affiliations:** 1 Department of Internal Medicine, Division of Cardiology, UT Southwestern Medical Center, Dallas, TX 75390 USA; 2 Laboratory of Regulatory Genomics, Cecil H. and Ida Green Center for Reproductive Biology Sciences, Division of Basic Reproductive Biology Research, Department of Obstetrics and Gynecology, University of Texas Southwestern Medical Center, Dallas, TX 75390 USA; 3 Department of Molecular Biology, UT Southwestern Medical Center, Dallas, TX 75390 USA; 4 McDermott Center for Human Growth and Development, UT Southwestern Medical Center, Dallas, TX 75390 USA; 5 Hamon Center for Regenerative Science and Medicine, Dallas, TX 75390 USA

## Abstract

The atrioventricular node (AVN) coordinates the timing of atrial and ventricular contraction to optimize cardiac performance. To study this critical function using mouse genetics, however, new reagents are needed that allow AVN-specific manipulation. Here we describe a novel Gjd3-CreEGFP mouse line that successfully recombines floxed alleles within the AVN beginning at E12.5. These mice have been engineered to express CreEGFP under the control of endogenous Gjd3 regulatory elements without perturbing native protein expression. Detailed histological analysis of Gjd3-CreEGFP mice reveals specific labeling of AVN cardiomyocytes and a subset of cardiac endothelial cells. Importantly, we show that Gjd3-CreEGFP mice have preserved cardiac mechanical and electrical function. In one application of our newly described mouse line, we provide a three-dimensional (3D) view of the AVN using tissue clearing combined with confocal microscopy. With this 3D model as a reference, we identify specific AVN sub-structures based on marker staining characteristics. In addition, we use our Gjd3-CreEGFP mice to guide microdissection of the AVN and construction of a single-cell atlas. Thus, our results establish a new transgenic tool for AVN-specific recombination, provide an updated model of AVN morphology, and describe a roadmap for exploring AVN cellular heterogeneity.

## Introduction

The heart is composed of multiple cell types that can be broadly categorized into cardiomyocytes (CMs) and non-CMs. In turn, CMs can be identified as primarily contractile or conducting. Together, conducting CMs comprise the cardiac conduction system (CCS), which coordinates the electrical impulse required for synchronized contraction of the heart^1–3^. Within the CCS, individual components perform specific functions. For example, specialized pacemaker (PM) myocytes in the sinoatrial node (SAN) initiate the electrical impulse, which is rapidly propagated throughout the atria^1–3^. These electrical impulses are then directed to the atrioventricular node (AVN), where a mandatory delay ensures completion of atrial contraction and ventricular filling prior to ventricular contraction^1–3^. In the adult murine heart, the AVN provides the only electrical connection between atrial and ventricular myocardium.

CCS structures are characterized by the presence of ion channels and gap junction proteins for efficient electrical conduction^4–7^. In the mouse heart, four major connexin (Cxs) proteins, Cx30.2, Cx40, Cx43, and Cx45, form gap junction channels to facilitate intracellular crosstalk^4–7^. The primary Cxs mediating low conductance cell-to-cell coupling in the SAN are Cx30.2 and Cx45^4–7^. In the AVN, gap junction channels formed by abundantly expressed Cx30.2, and less expressed Cx45 and Cx40 mediate cellular coupling. Cx30.2 and Cx45 provide higher intercellular resistance, thereby explaining the slower conduction velocity in the SAN and AVN compared to the VCS or working myocardium^4–7^. Mouse Cx30.2, also known as gap junction delta 3 (Gjd3) is a physiologically important marker of the AVN^8^. Cx30.2 preferentially labels the adult SAN and AVN, but not working myocardium of atria and ventricles, and genetic deletion of the Gjd3 gene results in accelerated conduction through the AVN^9^. Thus, we chose to target the Gjd3 locus in order to generate Gjd3^3′UTR-IRES-CreEGFP/+^ mice for labeling of AVN cells.

The AVN is structurally and molecularly complex. Early electrophysiology studies of the rabbit AVN established the existence of at least six cell types based on unique electrical properties^10^. In addition, structural complexity of the AVN was clearly demonstrated by three-dimensional (3D) reconstructions using both immunostaining^11^ and *in situ* hybridization data^12^. For example, these studies described key AVN sub-structures with specific markers, such as the transitional AV ring (Cx43^+^, Cx45^+^), nodal atrioventricular (AV) ring (Hcn4^+^, Tbx3^+^, Cx45^+^, Cx40^−^, Cx43^−^), compact AVN and inferior nodal extension (INE) (Hcn4^++^, Tbx3^+^, Cx45^++^, Cx40^−^, Cx43^−^), and AV bundle (AVB) and lower nodal cells (Hcn4^++^, Tbx3^+^, Cx45^++^, Cx40^+^, Cx43^−^). In recent years, however, newly described approaches for single-cell RNA sequencing^13^ and tissue clearing^14^ have improved the resolution of cellular and imaging studies, respectively. Thus, application of these contemporary methods may shed new light on the structural and functional complexity inherent to the AVN.

Here we describe a novel knockin (KI)-Cre reporter mouse model created by targeted homologous recombination of the Gjd3 gene that precisely labels the AVN while maintaining native gene expression. Using Rosa26-tdTomato reporter mice, we show that recombination mediated by Gjd3-CreEGFP co-localizes with well-established markers of the AVN beginning as early as E12.5. Importantly, we demonstrate that Gjd3^3′UTR-IRES-CreEGFP/+^ mice have normal contractile and electrical function. We use tissue clearing combined with confocal microscopy to provide an updated 3D model of the AVN. Gjd3-CreEGFP is expressed in both the compact and distal AVN structures with additional labeling of PECAM^+^ endothelial cells on the surface of the adult heart. Finally, we use Cre-driven reporter expression to guide dissection of AVN tissue for single cell RNA-seq (scRNA-seq) analysis, which reveals an unappreciated degree of cellular complexity residing within the P0 AVN. Based on the studies described, we anticipate that Gjd3^3′UTR-IRES-CreEGFP/+^ mice will enable important mechanistic studies to unravel AVN development and function, and we provide a starting point for the exploration of AVN cellular heterogeneity.

## Results

### Gjd3^3′UTR-IRES-CreEGFP/+^ mice recombine cells within the AV junction

**Figure 1:**
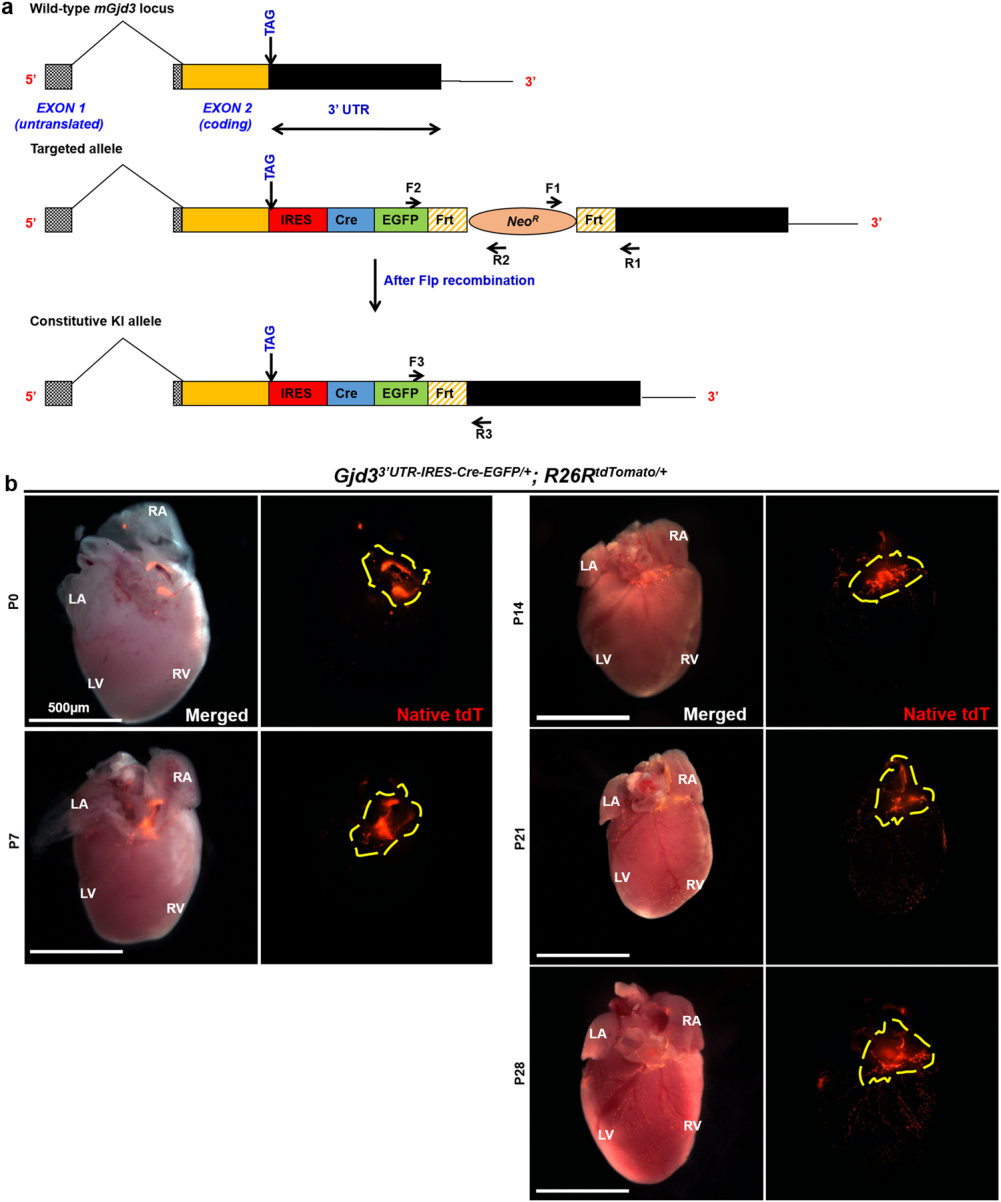
The Gjd3^3′UTR-IRES-CreEGFP/+^ allele mediates recombination in the postnatal AV junction. (**a**) Schematic of the endogenous Gjd3 locus (encoding for mouse Cx30.2) comprising untranslated exon 1 and exon 2, which contains the coding region (orange box) and 3′UTR (black box). A targeting vector was designed to insert an IRES-CreEGFP-FRT-Neo^R^-FRT cassette into the Gjd3 3′UTR. Following FLP-mediated recombination, CreEGFP is expressed under the control of the native regulatory sequences. PCR genotyping primer sets (F1-R1, F2-R2, and F3-R3) are indicated by black arrows, and the Gjd3 stop codon is shown. (**b**) Whole mount fluorescent images of hearts dissected from Gjd3^3′UTR-IRES-CreEGFP/+^; R26R^tdTomato/+^ mice at P0, P7, P14, P21, and P28. Robust tdTomato expression is observed at the AV junction (dashed outline). Number of hearts dissected (n) = 12 per group of animals; LA, Left Atrium; RA Right Atrium; LV, Left Ventricle; RV, Right Ventricle. Scale bar: 500 µm.

The mouse Gjd3 gene (GenBank accession number: NM_178596.2, Ensembl: ENSMUSG00000047197) is located on chromosome 11 and encodes for Connexin 30.2 (Cx30.2). The Gdj3 gene consists of an untranslated exon 1 followed by exon 2, which contains the entire coding region and 3′UTR (Fig. 1a). To target the Gjd3 locus for AVN labeling, a targeting vector was designed with 5′ and 3′ Homology Arms (HA) flanking an IRES-CreEGFP-FRT-Neo^R^-FRT cassette. Following homologous recombination, the cassette was precisely inserted in the 3′ UTR of the Gjd3 locus. This strategy was chosen to ensure unperturbed bicistronic expression of Gjd3 protein and a CreEGFP fusion protein under the control of the endogenous regulatory elements (Fig. 1a). Proper targeting of the knock-in cassette and FLP-mediated recombination was confirmed by PCR genotyping (Fig. S1).

To characterize the spatial expression of the Gjd^3′UTR-IRES-CreEGFP/+^ allele, Gjd3^3′UTR-IRES-CreEGFP/+^ mice were crossed with R26R^tdTomato/tdTomato^ reporter mice to generate Gjd3^3′UTR-IRES-CreEGFP/+^; R26R^tdTomato/+^ offspring (referred throughout the text as Cx30.2-tdTomato mice). We tracked tdTomato expression at various stages (P0, P7, P14, P21, P28) of postnatal murine growth in dissected hearts by epifluorescence microscopy. We observed robust and specific labeling at the dorsal AV junction in the vicinity of the presumptive AVN (Fig. 1b). We also observed reporter expression near the presumptive SAN (data not shown), which is consistent with previous reports^8^. tdTomato expression was observed in 12 independent Cx30.2-tdTomato mice from multiple litters and compared with 9 WT control littermates (Fig. S2). Interestingly, we also consistently observed tdTomato expression on the ventricular surface beginning at P14 (Fig. 1b).

Given that Gjd^3′UTR-IRES-CreEGFP/+^ mice express a CreEGFP fusion protein, we wished to determine whether the EGFP moiety was functional. We examined cryosections from several stages of development and postnatal maturation ranging from E12.5 to P42, and we were unable to detect native EGFP fluorescence (data not shown) or any signal following standard immunstaining procedures. Therefore, we sought to develop a method for detecting EGFP so that Gjd3^+^ (EGFP^+^) cells and Gjd3 lineage positive (tdTomato^+^) cells can be simultaneously detected in Gjd3^3′UTR-IRES-CreEGFP/+^; R26R^tdTomato/+^ mice. After testing several alternatives, we found that tyramide signal amplification (TSA) provided the best signal-to-noise ratio with minimal detectable background signal using P4 sections (Fig. S3). Interestingly, we observed a nearly 1:1 correlation between EGFP^+^ and tdTomato^+^ cells, suggesting minimal expansion of the Gjd3^+^ lineage in the postnatal heart (Figs S3 and S4).

**Figure 2:**
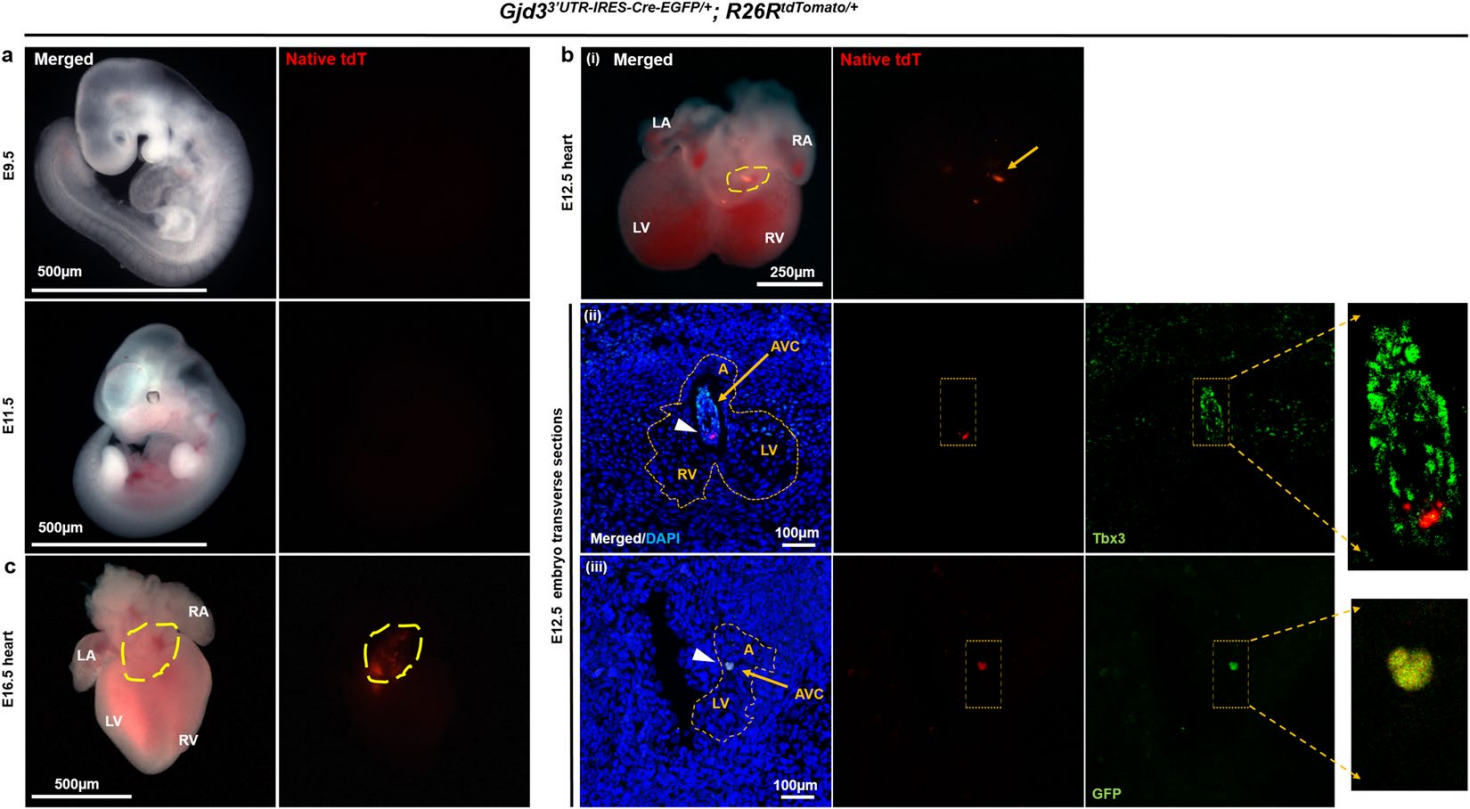
Gjd3-CreEGFP labels AVN progenitors beginning at E12.5. (**a**) Whole mount fluorescent images of E9.5 and E11.5 Gjd3^3′UTR-IRES-CreEGFP/+^; R26R^tdTomato/+^ embryos failed to demonstrate tdTomato expression. Number of hearts dissected (n) = 12 per group of animals; Scale bar: 500 µm. (**b**) (i) Heart from a E12.5 Gjd3^3′UTR-IRES-CreEGFP/+^; R26R^tdTomato/+^ embryo revealed bright reporter expression in the AVJ (dashed outline). (ii-iii) Transverse cryosections from an E12.5 Gjd3^3′UTR-IRES-CreEGFP/+^; R26R^tdTomato/+^ embryos were stained for (ii) Tbx3 (green) to demonstrate that tdTomato (red) localizes to the developing AVC (yellow arrow). (iii) Sister section was stained for GFP (IHC) (green) to confirm overlap with tdTomato (red) in the AVC (yellow arrow). Dashed yellow line outlines the developing heart, and white arrowheads indicate tdTomato^+^ cells. Zoomed insets are shown to the right for better visualization of signal overlap in the AVC. Nuclei were counterstained with DAPI (blue). (**c**) Whole mount fluorescent image of an E16.5 Gjd3^3′UTR-IRES-CreEGFP/+^; R26R^tdTomato/+^ embryo revealed persistent tdTomato reporter expression in the AVJ region (dashed outline). A, Atrium; LV, Left Ventricle; RV, Right Ventricle. Scale bars: as shown.

Since a Cx30.2-lacZ transgene is active as early as E8.5 during embryonic development^15^, we next evaluated tdTomato expression at various stages of embryogenesis. We did not observe any tdTomato expression in whole embryos at E9.5 or E11.5 (Fig. 2a), and serial sections through an E9.5 embryo failed to detect any tdTomato^+^ cells (data not shown). In contrast, E12.5 Cx30.2-tdTomato hearts displayed reporter expression in a discrete part of the AV junction (Fig. 2b, i). To confirm that tdTomato marked a subset of developing Cx30.2^+^ AVC cells, a Cx30.2-tdTomato embryo was cryosectioned. We performed immunostaining with antibodies to GFP, Cx30.2, and Tbx3, which marks the developing AVC beginning at E8.5^16^. High-power confocal images revealed that Cre-recombined tdTomato cells label a discrete subset of Tbx3^+^ embryonic AVC cells (Fig. 2b, ii). Furthermore, we found that tdTomato co-localizes with both GFP and Cx30.2 (Figs 2b, iii and S5) and that the subset of AVC cells marked by tdTomato at E12.5 was stable across multiple mouse hearts (data not shown). We also confirmed that Cx30.2-tdTomato expression persists in the AV junction at E16.5 (Fig. 2c), whereas sparse tdTomato labeling was observed in the right ventricle and outflow tract (Fig. S6). Taken together, these data demonstrate proper targeting of the Gjd3 locus with appropriate CreEGFP expression in the AV junction of embryonic and postnatal mice as early as E12.5. Interestingly, the restricted expression pattern of the Gjd3-CreEGFP knock-in line compared to our previous Cx30.2-lacZ transgenic line^15^ suggests that CreEGFP is more likely to accurately report endogenous Cx30.2 expression.

### Gjd3-CreEGFP marks the AVN and a subpopulation of endothelial cells

**Figure 3:**
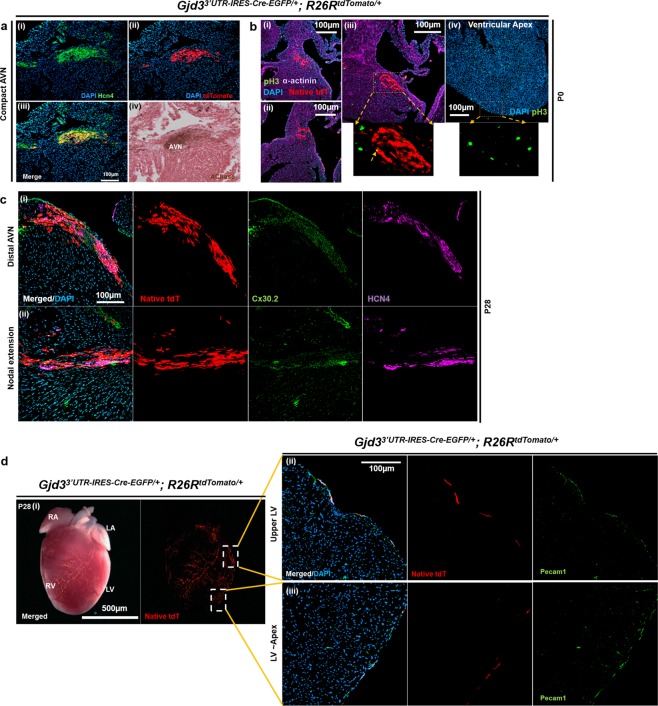
Gjd^3′UTR-IRES-CreEGFP/+^; R26R^tdTomato/+^ mice exhibit robust reporter expression in the atrioventricular node (AVN). (**a**) High-power confocal image of consecutive heart sections from a P0 Gjd3^3′UTR-IRES-CreEGFP/+^; R26R^tdTomato/+^ mouse were stained as follows: (i) Hcn4 (green), (ii) tdTomato (red), (iii) Merged (Hcn4/tdTomato overlap), and (iv) Acetylcholinesterase (AChase). (**b**) (i–iii) Consecutive heart cryosections from a P0 Gjd3^3′UTR-IRES-CreEGFP/+^; R26R^tdTomato/+^ mouse were co-stained with mitotic marker phospho-Histone 3 (pH3) and pan-cardiac marker sarcomeric α-actinin. Amongst the three AVN sections, only one pH3^+^tdTomato^+^ cells was identified (iii) (see inset and yellow arrow). (iv) In contrast, multiple pH3^+^ nuclei were identified in the ventricular apex (see inset). (**c**) Sections were obtained from a Gjd3^3′UTR-IRES-CreEGFP/+^; R26R^tdTomato/+^ mouse at P28 and stained for Cx30.2 (green) and Hcn4 (magenta). (i) In the distal AVN, almost complete overlap of tdTomato, Cx30.2, and Hcn4 was observed. (ii) In the nodal extension, similar overlap was seen. (**d**) (i) At P28, tdTomato reporter expression was also observed in cells along the surface of the heart. ii) PECAM-1 staining of cryosections confirmed co-localization of tdTomato in endothelial cells at the left ventricular (LV) free wall. (iii) Similarly, tdTomato marked endothelial cells at the LV apex based on PECAM-1 co-localization. Nuclei were counterstained with DAPI (blue). Scale bars: as shown for ((**a**–**d**(ii,iii)) 100 µm; (**d**(i)) 500 µm.

Although Gjd3-CreEGFP demonstrated proper anatomic localization within the heart, we next performed immunostaining on P0 heart cryosections from Cx30.2-tdTomato mice with specific markers to verify AVN labeling. From late fetal to early adult stages, HCN4 expression marks all components of the CCS^17^, and acetylcholinesterase (AChase) staining identifies all CCS components after birth^18^. We observed precise and complete co-localization of endogenous Hcn4 with tdTomato in the AVN (Fig. 3a, i–iii), confirming that the Gjd3^3′UTR-IRES-CreEGFP/+^ allele marks AVN cells of the conduction system. Moreover, we performed AChase staining on consecutive sections to further corroborate Cx30.2-tdTomato expression within the AVN (Fig. 3a, iv). In parallel, we crossed Gjd3^3′UTR-IRES-CreEGFP/+^ and Rosa26^lacZ/lacZ^ mice to generate Cx30.2-lacZ animals for staining with X-gal. These studies independently confirmed specific recombination within the AVN by the Gjd3^3′UTR-IRES-CreEGFP/+^ allele (Fig. S7).

Previous studies have established that AVN cells exit the cell cycle by mid-gestation during cardiac development^12,19,20^. In contrast, atrial and ventricular chamber CMs continue to proliferate after birth through the first week of life^21,22^. Therefore, we wished to confirm that Gjd3-CreEGFP^+^ cells were non-dividing AVN CMs. Therefore, we stained cryosections from P0 mice with a phospho-histone H3 (pH3) antibody to identify mitotically active cells (Fig. 3b, i–iii). We observed numerous pH3^+^ CMs in the interventricular septum (Fig. 3b, i–iii) and ventricular apex (Fig. 3b, iv), which is consistent with previous findings^21,22^. In contrast, we found only a single pH3^+^ AVN CM after analyzing multiple sections (Fig. 3b, i–iii and inset). These observations confirm that Gjd3-CreEGFP^+^ cells are lineage-committed AVN CMs that undergo infrequent cell division at P0, which is also consistent with prior studies^20^.

We next sought to confirm co-localization of tdTomato expression and endogenous Cx30.2 protein in Cx30.2-tdTomato mice at P28. By staining cryosections, we found complete overlap of endogenous Cx30.2 protein, HCN4, and tdTomato expression in both the distal AVN and AVN extension (Fig. 3c, i,ii). We also wished to verify that normal Cx30.2 protein levels were maintained in Gjd3^3′UTR-IRES-CreEGFP/+^ mice. Therefore, we performed Western blotting on whole heart tissue extract obtained from P42 Gjd3^3′UTR-IRES-CreEGFP/+^ and WT animals using antibodies against Cx30.2 and Hcn4 to demonstrate preserved protein expression (Figs S8a and S9). Quantification of the Western blots confirmed these observations (Fig. S8b). Furthermore, comparison of Cx30.2 immunostaining between Gjd3^3′UTR-IRES-CreEGFP/+^ and wild-type littermates revealed little change in Cx30.2 expression (Fig. S8c). Collectively, these studies show that Gjd3-CreEGFP labels the Cx30.2^+^/Hcn4^+^ AVN without perturbing endogenous Cx30.2 expression.

To establish the recombination efficiency in Gjd3-CreEGFP mice, we systematically compared tdTomato fluorescence with Cx30.2 staining in Gjd3^3′UTR-IRES-CreEGFP/+^; R26R^tdTomato/+^ mice at various stages of development from E12.5 to P42 (Fig. S10). From this series of immunostaining experiments, we quantified the recombination frequency based on the number of tdTomato^+^ cells divided by Cx30.2^+^ cells (Table S1). We found that the recombination efficiency was relatively consistent across developmental time points, ranging from 88% to 126%.

Because we observed that cells on the ventricular surface were also labeled with tdTomato (Fig. 3d, i), we wished to determine their identity. Since these tdTomato^+^ cells appeared to follow a vessel-like distribution, we hypothesized that they were endothelial cells. To test this hypothesis, we performed immunostaining with CD31/Pecam1 antibody, which is known to label vascular endothelium^23^. Confocal imaging revealed a high degree of overlap between tdTomato fluorescence and Pecam1 staining (Fig. 3d, ii,iii). In summary, these data demonstrate that the Gjd3-CreEGFP allele specifically labels the AVN and a subpopulation of cardiac endothelial cells.

### Gjd3^3′UTR-IRES-CreEGFP/+^ mice display normal contractile and electrical function

A major theoretical advantage of our KI-Cre allele is CreEGFP expression directed by Cx30.2 regulatory elements without perturbing endogenous protein expression. Since Gjd3 heterozygous mice could have mild conduction phenotypes, we sought to confirm phenotypic normalcy of Gjd3^3′UTR-IRES-CreEGFP/+^ mice. Overall, we found that litter sizes were normal and sex-distribution followed the expected Mendelian ratio (data not shown). Furthermore, the average lifespan of the KI-Cre animals was equivalent to WT littermates. Together, these observations suggest that the Gjd3 KI-allele does not have major deleterious consequences. Additionally, we performed Hematoxylin and Eosin (H&E) staining on P42 formalin-fixed heart sections from Gjd3^3′UTR-IRES-CreEGFP/+^ mice and control littermates to confirm that overall cardiac morphology was unaltered (Fig. S11).

**Figure 4:**
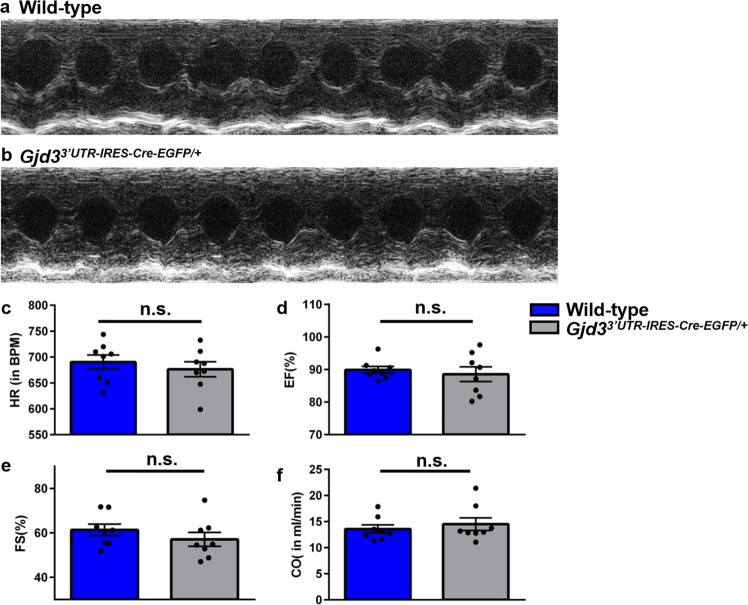
Gjd3^3′UTR-IRES-CreEGFP/+^ mice maintain cardiac mechanical function. (**a**) Representative M-mode tracing of a wild-type mouse at P42. (**b**) Representative M-mode tracing of a Gjd3^3′UTR-IRES-CreEGFP/+^ mouse at P42. Based on M-mode echocardiography on wild-type and Gjd3^3′UTR-IRES-CreEGFP/+^ mice (n = 8 in each group), the following parameters were calculated: (**c**) Heart Rate (HR) in Beats Per Minute (BPM) was calculated, (**d**) Ejection Fraction (EF) in percentage, (**e**) Fractional Shortening (FS) in percentage, and (**f**) Cardiac Output (CO) in milliliters per minute (ml/min). Black circles represent individual WT and Gjd3^3′UTR-IRES-CreEGFP/+^ mice used in the study. Blue bars and grey bars represent WT and Gjd3^3′UTR-IRES-CreEGFP/+^ mice, respectively. No statistically significant differences (p-value > 0.05; labeled as n.s. [not significant]) were observed between groups.

Next, we sought to determine whether Gjd3^3′UTR-IRES-CreEGFP/+^ mice develop cardiac mechanical dysfunction. In order to evaluate cardiac mechanical parameters in Gjd3^3′UTR-IRES-CreEGFP/+^ mice, we performed M-mode echocardiography (Fig. 4a,b) on awake P42 WT and Gjd3^3′UTR-IRES-CreEGFP/+^ mice (n = 8 per group). We measured Heart Rate (HR), Ejection Fraction (EF), Fractional Shortening (FS), and Cardiac Output (CO) in both groups of animals (Fig. 4c–f). Statistical testing on the two groups of animals revealed no significant differences (p-value > 0.05). Other heart function parameters, like Stroke Volume, Left Ventricular Mass, Systolic and Diastolic Diameter and Volume, were also calculated and appeared unchanged (data not shown).

**Figure 5:**
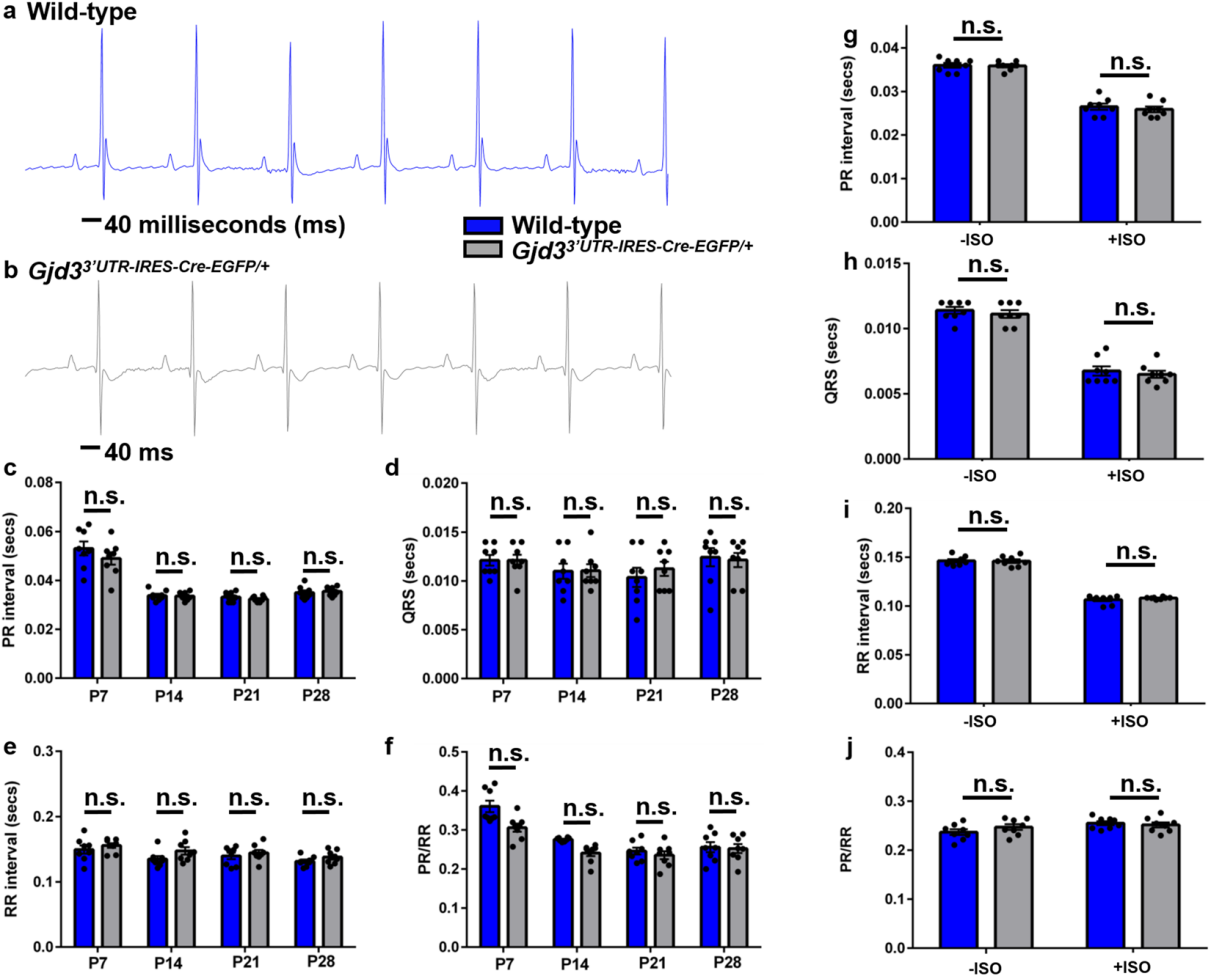
Cardiac electrical properties are preserved in Gjd3^3′UTR-IRES-CreEGFP/+^ mice. (**a**) Representative electrocardiogram (ECG) tracing of a wild-type mouse at P42. (**b**) Representative ECG tracing of a Gjd3^3′UTR-IRES-CreEGFP/+^ mouse at P42. Based on ECGs obtained from wild-type and Gjd3^3′UTR-IRES-CreEGFP/+^ mice (n = 8 in each group) at P7, P14, P21, and P28, the following parameters were measured: (**c**) PR interval (in seconds [secs]), (**d**) QRS width (in secs), (**e**) RR interval (in secs), and (**f**) PR/RR ratio At P28, mice in both groups were subjected to isoproterenol stimulation, and the following parameters were measured before and after stimulation: (**g**) PR interval (in secs), (**h**) QRS width (in secs), (**i**) RR interval (in secs), and (**j**) PR/RR ratio. Black circles represent individual WT and Gjd3^3′UTR-IRES-CreEGFP/+^ mice used in the study. Blue bars and grey bars represent WT and Gjd3^3′UTR-IRES-CreEGFP/+^ mice, respectively. No statistically significant differences (p-value > 0.05; labeled as n.s. [not significant]) were observed between groups.

To demonstrate that electrical parameters are unaffected in Gjd3^3′UTR-IRES-CreEGFP/+^ mice, we recorded serial surface lead II ECGs on both groups of mice (n = 8 per group) under mild anesthesia at P7, P14, P21, and P28. ECG traces appeared normal in both WT and Gjd3^3′UTR-IRES-CreEGFP/+^ mice (Fig. 5a,b). From independent ECG traces, we also measured PR interval, RR interval, QRS width, and PR/RR ratio. We observed that electrical activity was similar in both groups without any statistically significant differences across all monitored developmental stages (Fig. 5c–f). We also assessed cardiac electrical function after mice were subjected to a stress regime. We administered Isoproterenol (ISO) (300 µg of ISO/20 g animal body weight) intra-peritoneally in P28 KI-Cre (n = 8) and WT (n = 8) mice. ECGs were recorded pre- and post-ISO injection using an established protocol^24^. We observed the expected PR interval shortening due to ISO administration (Fig. 5g). Importantly, there was no statistically significant difference in the electrical parameters (including RR interval, QRS width, and PR/RR ratio) between the two groups (Fig. 5g–j). Given that homozygous Cx30.2 knockout (KO) animals show a modest increase in PQ interval and AH interval^7^, we measured electrical activity in adult homozygous KI-Cre animals (n = 3) and observed normal AV conduction as indicated by the measured PR interval (Fig. S12). Collectively, these studies show that Gjd3^3′UTR-IRES-CreEGFP/+^ mice have normal cardiac mechanical and electrical function. Furthermore, preliminary data indicates that homozygosity of the Gjd3-CreEGFP allele has a minimal effect on AV conduction, but conclusive demonstration will require additional studies.

### Gjd3-CreEGFP outlines AVN morphology and labels specific AVN cardiomyocytes

**Figure 6:**
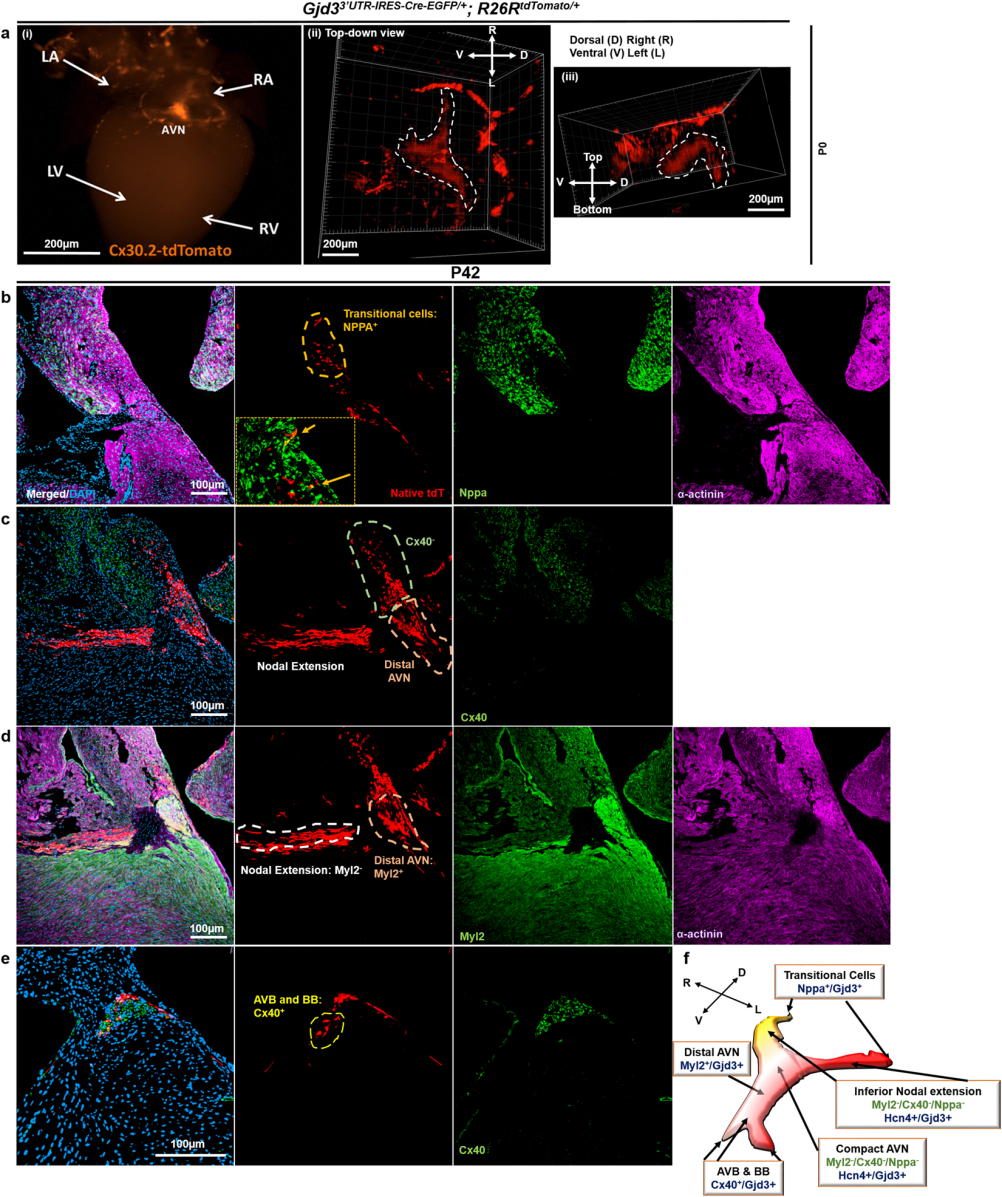
Cx30.2-tdTomato mice permit morphological characterization of the AVN. (**a**) (i) The heart of a P0 Gjd3^3′UTR-IRES-CreEGFP/+^; R26R^tdTomato/+^ (Cx302-tdTomato) mouse was cleared by CUBIC (See Methods section) and imaged by whole-mount fluorescence microscopy. Confocal microscopy was used to image the AVN from the cleared heart and to provide a three-dimensional model, and selected snapshots (see Supplementary Video) are shown from the following views: (ii) top-down and (iii) end-on. A compact node with bilateral inferior nodal extensions and a single distal nodal extension can been observed in the Cx30.2^+^ AVN image. (**b**) The heart of a P42 Gjd3^3′UTR-IRES-CreEGFP/+^; R26R^tdTomato/+^ mouse was sectioned through the AVN and stained for Nppa (green) and α-actinin (magenta). See inset for visualization of tdTomato^+^/Nppa^+^ atrial-nodal transitional cells. (**c**) A more distal section containing an inferior nodal extension and the distal AVN was stained for Cx40 (green). (**d**) A consecutive section was stained for Myl2 (green) and α-actinin (magenta). (**e**) A distal section through the AV bundle (AVB) was stained for Cx40 (green). (**f**) AVN model based on 3D morphology observed in (**a**) with individual anatomical segments labeled with corresponding markers. Nuclei were counterstained with DAPI. A coordinate plane with labeled directions relative to the heart specimen are provided for orientation in (**a**) (ii,iii) and (**f**). Scale bars: as shown.

To better appreciate the morphology of the Cx30.2-tdTomato^+^ expression domain, we cleared a P0 heart by CUBIC^14^ followed by whole-mount epifluorescence imaging (Fig. 6a, i). Serial images were obtained by confocal microscopy to render a 3D model of the Cx30.2^+^ AVN (Video 1). Top-down and end-on stillshots from the video rendering of the Cx30.2-tdTomato^+^ AVN (Fig. 6a, ii,iii) clearly demonstrate its unique morphology. Interestingly, we observe that the Gjd3-tdTomato^+^ AVN exhibits a dorsal compact nodal structure with bilateral inferior nodal extensions that project posteriorly and a distal region that extends anteriorly towards the ventricles (Fig. 6a, ii,iii).

Previous studies have established elegant 3D models of the atrioventricular conduction axis with specific markers of individual sub-compartments^11,12^. Given that the Cx30.2-tdTomato reporter enabled three-dimensional reconstruction of the Cx30.2^+^ AVN, we wished to evaluate specific markers with reference to the 3D model. For these studies, we co-localized tdTomato with the following markers: Nppa to label atrial CMs^25^, Myl2 to label ventricular CMs^26^, and Cx40 to label atrial and VCS CMs^27^. Consistent with previous reports, we observe little overlap between tdTomato and Nppa in the AVN aside from a few transitional cells (Fig. 6b and inset). Importantly, all AVN cells labeled by tdTomato co-localize with α-actinin, confirming that they are cardiomyocytes (Fig. 6b). Interestingly, neither the inferior nodal extension nor distal AVN Cx30.2-tdTomato^+^ cells appear to co-localize with Cx40 (Fig. 6c), suggesting that the latter cells comprise the lower nodal cells rather than the proximal AV bundle^12^. Furthermore, we observed that recombined tdTomato^+^ cells in the proximal AVN are devoid of Myl2 staining, while the distal portion of the AVN co-localizes with Myl2 (Fig. 6d). Finally, we found that Cx30.2-tdTomato labels a subset of proximal AVB and bundle branch cells as assessed by co-localization with Cx40 (Fig. 6e). We synthesized our 3D imaging (Fig. 6a) and immunostaining data (Fig. 6b–e) to derive a summary model for the AVN (Fig. 6f).

### Gjd3-CreEGFP guides establishment of a single-cell atlas for the AVN

**Figure 7:**
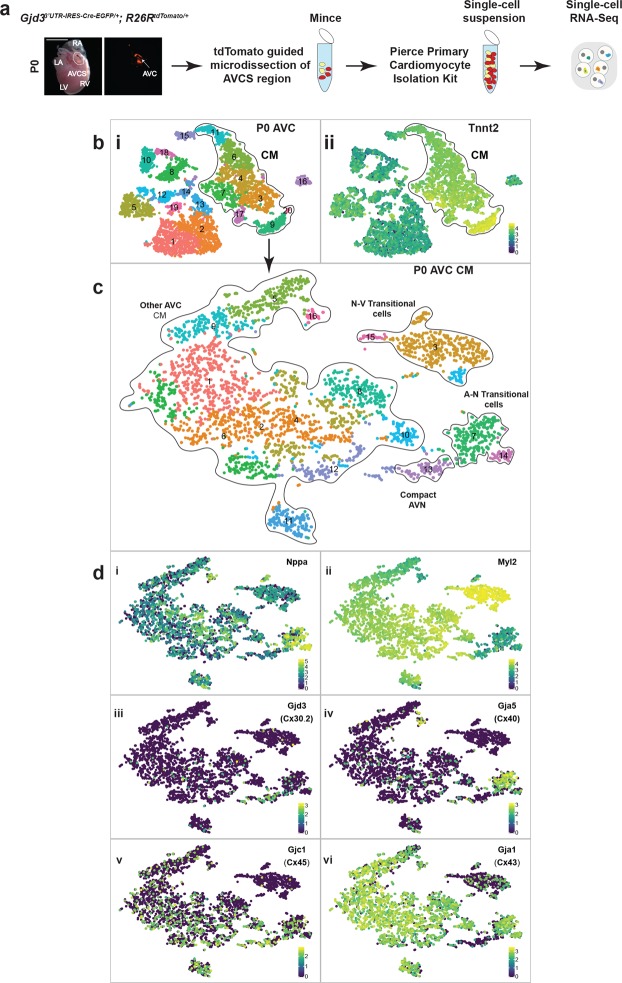
Gjd^3′UTR-IRES-CreEGFP/+^ mice enable creation of a single cell atlas for the AVN. (**a**) Schematic showing micro-dissection of P0 Gjd3^3′UTR-IRES-CreEGFP/+^; R26R^tdTomato/+^ mouse hearts based on tdTomato expression. A single-cell suspension was created from the dissected AVC tissue and subjected to single-cell RNA sequencing (scRNA-seq) analysis using the 10X Genomics pipeline. (**b**) (i) A t-distributed stochastic neighbor embedding (t-SNE) plot was generated for P0 AVC cells. Cardiomyocytes (CMs) were identified (dotted outline) based on Tnnt2 expression (ii). (**c**) P0 non-CMs were removed, and the remaining AVC CMs were re-clustered and visualized by t-SNE plot. The 16 identified clusters were grouped into 4 meta-clusters (dotted outline) based on shared and unique markers. (**d**) t-SNE plots of P0 AVC CMs with superimposed marker gene expression: (i) Nppa, (ii) Myl2, (iii) Cx30.2, (iv) Cx40, (v) Cx45, and (vi) Cx43. AVC, atrioventricular canal; CM, cardiomyocyte; A-N, atrial-nodal; N-V, nodal-ventricular. Colored scale in (**b)** (ii) and (**d**) (i–vi): Expression of transcripts in log2 scale; darker color indicates low or no expression and lighter color indicates high expression.

Recent advances in sequencing technology have made it possible to profile the transcriptome of single cells. Given that the cellular composition and heterogeneity of the AVN remains to be fully understood, we sought to establish a single cell AVN atlas. Therefore, we took advantage of our Cx30.2-tdTomato mice to guide microdissection of the P0 AVN (Fig. 7a). Using this strategy, we digested AVN-containing tissue into a single-cell suspension and conducted scRNA-seq analysis using the 10X Genomics platform. Applying t-Distributed Stochastic Neighbor Embedding (t-SNE) to the dataset, we identified 20 cell clusters, including CMs, fibroblasts, epicardial cells, immune cells, and other cell types (Fig. 7b). In order to better separate individual AVN CMs, we eliminated Tnnt2^−^ non-myocytes and re-clustered the remaining cells (Fig. 7c). This analysis demonstrated 16 clusters of AVC CMs at P0. As a point of reference, we mapped the expression of known AVN marker genes (Hcn4, Tbx3, and Tbx2) onto the AVN CM cell atlas (Fig. S13). Using supervised analysis with established chamber and CCS markers (Fig. 7d), we assigned 4 meta-clusters: (1) compact AVN cells (Cx30.2^+^/Cx45^+^/Cx40^−^/Cx43^−^/Nppa^−^/Myl2^−^), (2) atrial-nodal (A-N) transitional cells (Cx30.2^+^/Cx43^+^/Cx40^+/−^/Nppa^+^/Myl2^−^), (3) nodal-ventricular (N-V) transitional cells (Cx40^−^/Cx45^−^/Cx43^−^/Nppa^−^/Myl2^+^), and (4) other AVC CMs. Collectively, these results establish a single cell atlas for the P0 AVN using Gjd3^3′UTR-IRES-CreEGFP/+^ mice.

## Discussion

Here we characterize a new Gjd3 allele engineered by targeted insertion of an IRES-CreEGFP cassette into the 3′UTR of the endogenous locus. We show that Gjd3-CreEGFP mice precisely label the AVN beginning at E12.5 and a population of cardiac endothelial cells in adult mice. Importantly, we demonstrate that the levels of endogenous cardiac markers remain unaltered in Gjd3^3′UTR-IRES-CreEGFP/+^ mice and that cardiac mechanical and electrical functions are preserved. We provide an updated 3D reconstruction of the AVN and show that Gjd3-CreEGFP is expressed in both the compact and distal AVN structures in specific cardiomyocyte subsets. Finally, we use Gjd3^3′UTR-IRES-CreEGFP/+^ mice to establish a single cell atlas of the P0 AVN. We anticipate that the Gjd3-CreEGFP mouse line will serve as a powerful *in vivo* genetic reagent to investigate AVN lineage specification and function.

Tissue-specific Cre mouse lines have accelerated our understanding of CCS specification and function. In particular, KI-Cre lines most faithfully recapitulate the pattern of native gene expression^24,28–31^. Most KI strategies replace the native gene coding exons with a Cre cassette in the endogenous locus^28,29,32^, but haploinsufficiency of functionally important genes can lead to significant conduction phenotypes^32,33^. To overcome this potential issue, an IRES cassette was used to express Cre under the control of native regulatory elements while preserving expression of the endogenous gene. We have previously used this approach to generate a Cntn2-CreEGFP mouse line that directs expression of Cre to the ventricular conduction system (VCS) yet preserves normal Cntn2 expression and cardiac function^24^.

Several features of Gjd3^3′UTR-IRES-CreEGFP/+^ mice distinguish it from previously described mouse lines. First, since Gjd3 marks lineage-committed AVN CMs, Gjd3-CreEGFP will be particularly useful for assessing the function of genes within the AVN compartment especially after birth. Although the Tbx3-Cre line is well-suited for studying gene function in AVN progenitors, adult phenotypes are potentially complicated by ventricular CMs that descend from the Tbx3^+^ lineage^34^ if tamoxifen induction is carried out before E13-14. Second, Gjd3^3′UTR-IRES-CreEGFP/+^ mice demonstrate preserved Gjd3 protein levels and cardiac function. Because the IRES-CreEGFP cassette does not require haploinsufficiency, any phenotypes observed by conditional knockout can be entirely attributed to the gene of interest. Thus, we believe that Gjd3-CreEGFP mice can be used to study the tissue-specific function of individual genes in the least confounding context and should facilitate studies aimed at the mechanistic underpinnings of AVN formation and function. However, we do note that the utility of Gjd3-CreEGFP mice might be limited during embryonic development due to its late and restricted expression.

Recent studies have clearly shown that individual cell types can be further sub-categorized based on scRNA-seq analysis^13^. Here we use our Gjd3^3′UTR-IRES-CreEGFP/+^ mice to guide AVN dissection and establishment of a single cell atlas. We identified 16 distinct CM subtypes that exist within the AVN. Interestingly, prior studies defined at least six cell types in the rabbit AVN based on unique electrical properties^35^. In the future, it will be important to classify newly identified AVN cell types based on specific ion channel and/or gap junction repertoires. We envision that cell types distinguished by transcriptomic signatures can then be verified by electrophysiological studies. In addition, it will be of great interest to determine the gene regulatory networks responsible for this high degree of AVN cell functional heterogeneity.

Previous models of AVN morphology have been based on inference or 3D reconstructions from tissue sections. Here we provide a model based on confocal imaging of a whole heart specimen, which provides direct visualization of AVN morphology without reconstruction. Interestingly, many features of our 3D model and single-cell atlas are consistent with previous observations. For example, the bipartite inferior nodal extension identified in our 3D AVN model (Fig. 6a) is consistent with prior reconstructions from both mouse and human tissues^12,36^. Interestingly, the nodal extensions represent potential conduits for the slow and fast pathways that mediate AVN re-entrant tachycardia (AVNRT)^12,37^. Furthermore, synthesizing the information from our 3D AVN model with the AVN single cell atlas provides additional novel insights. For example, individual AVN clusters express unique combinations of connexins (Fig. 7d), and such heterogeneity may contribute to the electrical heterogeneity postulated to establish slow and fast AV nodal pathways^38^. In the future, it will be important to follow up on these observations in more detail. For these future investigations and many other applications, we anticipate that our Gjd3^3′UTR-IRES-CreEGFP/+^ mice will be broadly applicable for a variety of mechanistic studies.

## Methods

### Generation of the Knock-In (KI) Reporter Gjd3^3′UTR-IRES-CreEGFP/+^ Mice

The targeting vector contained an IRES-CreEGFP-FRT-NeoR-FRT KI cassette flanked by Homology Arms (HA) to ensure efficient Homologous Recombination (HR) within the 3′ UTR of the endogenous mouse Gjd3 locus. Standard methods were used to engineer genetically modified mice (Cyagen Biosciences Inc., Santa Clara, CA). To engineer the targeting vector, homology arms were generated by PCR using BAC clone RP23-313C22 or RP23-333D2 from the C57BL/6 J library as template. In the targeting vector, DT-A was used for negative selection (not shown). C57BL/6 mouse Embryonic Stem (mES) cells were used for gene targeting by standard electroporation technique. The ES cell clones with correct HR were selected for by Neomycin (G418) resistance (NeoR) and screened by Polymerase Chain Reaction (PCR). ES cells were expanded, and injected blastocysts were implanted into pseudo-pregnant mice. We used standard Polymerase Chain Reaction (PCR) screening to demonstrate (1) correct HDR using primer set F1-R1 designed against NeoR and junction of the starting of the Gjd3 3′UTR. The expected 632 bp fragment indicated the wildtype allele and 495 bp fragment the recombinant allele; (2) correct insertion of the KI allele using primer set F2-R2 designed to amplify a 546 bp product from the 3′ end of the KI cassette and 5′end of the right HA, (3) NeoR cassette removal (positive selection) with primer sets F3-R3 designed such that a band of 372 bp is detected upon NeoR deletion mediated by Flp, and (4) the Cre sequence with primer sets F4-R4 that amplify ~410 bp genomic fragment (Fig. S1b–d). Finally, we performed high fidelity PCR coupled with Sanger sequencing of the 3′ KI cassette boundary to conclusively show appropriate genomic targeting and the fidelity of the recombined junctions by Sanger sequencing (Fig. S1e). Primer sequences are provided in Table S2.

### Commercial Mouse Strains

The FLP deleter strain (Strain number 009086) [24], the R26R^tdTomato/tdTomato^ reporter (Strain number 007914)^39^, and R26R-LacZ^40^ [from Induced Mutant Resource of the Jackson Laboratory (stock numbers 3309 and 3310)] mice were obtained from the Jackson Laboratory (Bar Harbor, ME). For identification of the FLP allele and the *R26R* allele, we used two distinct primer sets (labelled Flp and R26R) which are enlisted in Supplementary Table S2. The newly generated KI reporter strain was maintained in a C57BL/6 background. All animal procedures were approved by the Institutional Animal Care and Use Committee at UT Southwestern Medical Center. All experiments and methods were performed in accordance with regulations set forth by the National Institutes of Health mandate for the care and use of laboratory animals.

### Cloning

We performed high-fidelity PCR using Phusion DNA Polymerase (Thermo Scientific, #F-530S) to amplify the 3′ KI cassette boundary with primer set F3-R3 to ensure appropriate genomic targeting and the fidelity of the recombined junctions. Next, the PCR fragment was cloned into pCR™2.1 cloning vector and performed Blue-white screening. The white colonies were used to obtain plasmid DNA followed by Sanger sequencing of the KI cassette boundary with M13-Forward primer (Sequence: Supplementary Table S2).

### Epifluorescent Microscopy

Equipment and Settings: Whole mount fluorescent images of whole embryos and organs were acquired with Zeiss Stemi SV11 dissection microscope equipped with epifluorescent and bright field illuminators and Optronics Macrofire camera setup. Objectives used were 0.63X and 1.6X, and scale bars were drawn based on the objective and zoom factor used.

### Modified CUBIC (clear, unobstructed brain imaging cocktails and computational analysis) Clearing Protocol

P0 Gjd3^3′UTR-IRES-CreEGFP/+^; R26R^tdTomato/+^ mouse heart was dissected and washed in PBS, fixed in 4% PFA in PBS for 30 minutes at 4 °C, followed by two successive washes with PBS for 10 minutes each. Next, the fixed heart was immersed in 1 ml of CUBIC- Reagent 1^14^ and gently shaken at 37 °C for 3 days. At this point, the incubating CUBIC- Reagent 1 is replaced with fresh CUBIC- Reagent 1 for an additional 1-2 days or until the tissue looks almost transparent, up to a week at the most. The treated heart was washed with PBS several times at room temperature (RT) while gently shaking, immersed in 20% sucrose in PBS, degassed, and immersed in CUBIC- Reagent 2^14^ at 37 °C for 7 days. The tissue was washed 5 times with PBS-Tween-20 (0.1%) solution for 30 minutes each at RT. The cleared heart was stored in 3% BSA- PBS-Tween-20 (0.1%) at 4 °C wrapped in parafilm until imaged. The CUBIC cleared heart was processed in 30% sorbitol, 50% sorbitol, and 70% sorbitol at RT for 30 minutes with gentle shaking for each of the gradients. The heart was finally immersed in 70% sorbitol in a glass chamber and imaged using confocal microscopy.

### Antibodies

The following primary antibodies were used [species, target, dilution, company and/or product number]: rabbit anti-Cx40, 1:250 (Alpha Diagnostic International); rabbit polyclonal anti-Cx30.2 (LifeTechnologies, #40-7400), 4 µg/ml for IF staining and 1 µg/ml for WB analysis; mouse mAb α-Tubulin (DM1A) (Cell-Signaling Technology, #3873), 1:1000 for WB analysis; guinea-pig polyclonal anti-Hcn4 (Alomone Labs, #AGP-004), 1:200 for IF; rabbit polyclonal anti-Hcn4, 1:200 for WB analysis, (Alomone Labs, #APC-052); rabbit polyclonal anti-Tbx3 (abcam, #ab99302), 5 µg/ml for IF staining; Pecam1/CD31 Rat anti-Mouse, Unlabeled, Clone: MEC 13.3 (BD Biosciences, #BDB553370), 1:50; rabbit polyclonal anti-Nppa (Millipore, #AB5490), 1:100; mouse monoclonal anti-sarcomeric actinin (Sigma, #A7811), 1:100; rabbit polyclonal anti- Myl2 antibody, 1:100 (ProteinTech); and chicken polyclonal anti-GFP antibody, 1:100 (Thermo Fisher Scientific, #A10262). The following fluorophore-conjugated secondary antibodies were used: Alexa Fluor 488 Goat Anti-Rabbit IgG (H + L) Antibody, 1:400 (Invitrogen); Alexa Fluor 488 Rabbit Anti-Mouse IgG (H + L) Antibody, 1:400 (Invitrogen); Alexa Fluor 488 Rabbit Anti-Goat IgG (H + L) Antibody, 1:400 (abcam); Alexa Fluor 647 goat anti-guinea pig IgG (H + L) highly cross-adsorbed secondary antibody, 1:400 (Invitrogen); Alexa Fluor 647 Donkey Anti-Mouse IgG (H + L) Antibody, 1:400 (Invitrogen). Antibodies were routinely diluted in 1X Universal Blocking Reagent (Biogenex Laboratories) at the appropriate concentration except for Cx30.2 antibody (described in details under Immunostaining).

### Immunostaining

Freshly dissected mouse hearts were fixed in 4% PFA at 4 °C, then equilibrated in 10% sucrose, followed by 20% sucrose overnight at 4 °C and embedded in Tissue Freezing Medium. 8 µm thick cryosections were acquired followed by permeabilization for 20 minutes in 0.3% Triton X-100. Next, the sections were blocked for 10 minutes at 25 °C in 1X Universal Blocking Reagent. (In particular, for Cx30.2 antibody staining, 4% Normal Goat Serum (NGS) (Vector Labs, S-1000) in 1X PBS was used as the blocking reagent and for primary antibody dilution). Slides were then incubated overnight at 4 °C with the primary antibody followed by incubation for 1 hour at 25 °C in the dark with the appropriate combination of secondary antibody and mounted with Vectashield with the nuclear counter stain DAPI (Vector Labs). All steps were performed in a dark humidified chamber.

### Immunohistochemistry (IHC)

Similar to immunostaining, 8 µm thick cryosections were acquired on glass slides. This was followed by permeabilization for 20 minutes in 0.3% Triton X-100 in 1X PBS. Next, the sections were washed three times with 1X PBS for 5 minutes each. The sections were treated with 3% hydrogen peroxide (H_2_O_2_) (Stock Hydrogen Peroxide solution, 30%, Sigma, #H1009) solution made in PBS for 10 minutes at 25 °C followed by two PBS washes, 5 minutes each. Sections were blocked for 10 minutes at 25 °C in 1X Universal Blocking Reagent. Slides were then incubated overnight at 4 °C with the chicken anti-GFP primary antibody. Upon washing three times with 1X PBS, sections were incubated for 1 hour at 25 °C with Pierce Goat anti-Chicken IgY, H and L Secondary Antibody, Horse Radish Peroxidase (HRP) conjugate (Thermo Scientific, #PA1-28658, 1:200) diluted in 1X Universal Blocking Reagent. While rinsing off the secondary antibody with 1X PBS, fresh tyramide solution was prepared by diluting the tyramide stock (Labeled Tyramide Alexa Fluor® 488, #T20948) solution 1:100 in amplification buffer/0.0015% H_2_O_2_ (Components from Tyramide Signal Amplification Kit from Molecular Probes was used) just prior to labeling. The sections were incubated with working Tyramide solution for 10 minutes at 25 °C. The slides were rinsed with 1X PBS for three times and then mounted with Vectashield with the nuclear counter stain DAPI. All steps were performed in a dark humidified chamber.

### Western Blot (WB) Analysis

For tissue samples, whole hearts were dissected from n = 3 independent P42 Gjd3^3′UTR-IRES-CreEGFP/+^ and WT mice and immediately cryopreserved in liquid nitrogen. Cryofrozen heart samples were then pulverized and homogenized in pre-chilled RIPA buffer (50 mM Tris, pH 7.5, 150 mM NaCl, 1% NP-40, 0.5% sodium deoxycholate, 0.1% sodium dodecyl sulfate, protease and phosphatase inhibitors, make up volume with sterile water). Further tissue disruption was carried out by three cycles of alternate dry ice and wet ice incubation for 10 minutes each. The homogenate was centrifuged at 1000 × g for 5 minutes at 4 °C. The supernatant was used as the total tissue lysate. For estimation of concentration obtained upon protein extraction from the samples, we used Pierce Coomassie Plus (Bradford) Assay Kit (Thermo Fisher Scientific). Samples were run on a 6% denaturing polyacrylamide gradient gel and transferred to PVDF membrane (Bio-Rad) overnight at 4 °C. PVDF membrane was blocked the following day with 5% BSA in PBS with Tween-20 (0.1%) at room temperature for 1 hour. Membrane was then incubated with specific primary antibodies diluted in the same blocking reagent overnight at 4 °C followed by wash steps and HRP-conjugated secondary antibodies for chemiluminescent detection of target antigen-antibody complexes after incubation of membrane with Pierce™ ECL Western Blotting Substrate (ThermoFisher Scientific, 32106).

#### Equipment and Settings

LI-COR Odyssey Fc Imaging system was used to image the ECL blots at 425 nm emission. Image Studio™ Lite Ver 5.2 was used to analyze data and import images in.tif file formats. Image display was adjusted without changing the raw data. Preliminary quantification was based on signal intensities of bands using Image Studio™ Lite Ver 5.2. The level of protein expression between two groups was finally quantified using ImageJ software plugin and plotted using GraphPad Prism 6 software.

### Acetylcholinesterase Histochemistry

For Acetylcholinesterase histochemical staining, we followed the protocol that has been described previously^24^. Equipment and settings: Images were captured using Leica DM2000 upright compound microscope with Optronics Microfire camera. Objectives used were 10X and 20X.

### Histology

Freshly dissected hearts were fixed overnight in 10% Neutral Buffered Formalin. Routine Hematoxylin and Eosin (H&E) staining was performed on paraffin-embedded heart sections. Equipment and settings: Images were captured using Leica DM2000 upright compound microscope with Optronics Microfire camera. Objective used was 1.25X.

### X-gal staining of whole hearts

Freshly dissected whole hearts were fixed in freshly prepared 0.2% glutaraldehyde solution (Stock 8%) in 1X PBS at 4 °C with gentle shaking in a 24 well plate for 1 hour. Fixative is removed, and the hearts are washed twice with wash solution (prepared fresh; Total volume prepared is 3 times in excess: Stock 10X PBS, 0.01% Deoxycholic acid (Stock 10% (100 mg/ml), 0.02% NP-40 (=Igepal) (Stock 100%), double distilled water to make up the volume such that final concentration is 1X PBS) for 10 minutes each at 25 °C. The staining solution stock components (0.5 M Potassium Ferricyanide (K_3_Fe(CN)_6_) in 1X PBS wrapped in foil and stored at 25 °C, 0.5 M Potassium Ferrocyanide (K_4_Fe(CN)_6_) in 1X PBS wrapped in foil and stored at 25 °C, 40 g/ml X-gal prepared in Dimethylformamide (DMF) and stored at −20 °C, 1 M Magnesium Chloride (MgCl_2_)) can be prepared and stored at recommended temperatures. The working staining solution was prepared fresh (For a volume of 20 ml of the X-gal staining solution, 200 µl K_3_Fe(CN)_6_, 200 µl K_4_Fe(CN)_6_, 500 µl 40 g/ml X-gal, 40 µl 1 M MgCl_2_, and 19 ml of wash solution). The washed hearts were submerged in the staining solution, the plate was wrapped in foil, and incubated overnight at 25 °C with gentle shaking. The staining solution was removed next day and washed with 1X PBS thoroughly. The hearts were checked under microscope to ensure staining. The hearts were post-fixed overnight in 10% NBF at 4 °C with gentle shaking. Next day, the fixative was removed and submitted to histology core in 1X PBS for four-chamber view sectioning ensuring preservation of LacZ and Nuclear Fast Red (NFR) counterstaining. Equipment and settings: Images were captured using Leica DM2000 upright compound microscope with Optronics Microfire camera. Objectives used was 20X.

### Confocal Microscopy

Equipment and settings: Confocal images of immunostained heart sections were acquired using Nikon A1R+ scanning confocal system. Objectives used were 10X and 20X (dry). Excitation laser filters used were 488 nm line; green fluorescence, 555 nM line; red fluorescence, and 647 nm line; far-red fluorescence. Images were analyzed using NIS Elements Viewer v4.2 software, ImageJ and Adobe Photoshop CS6 Extended software.

### Echocardiography

Equipment and settings: Vevo2100 high-resolution digital imaging platform was used to evaluate the mechanical properties of P42 hearts from both groups of animals by M-mode echocardiography in unanesthetized animals. VevoStrain™ Analysis software was used to process the data and calculate the heart function parameters like Heart Rate, Fractional Shortening, etc.

### Electrocardiography (ECG or ECG)

Serial surface ECG was performed on both groups of mice under anesthesia (2% isoflurane in 200 mL/min oxygen) throughout different developmental stages. The subcutaneous leads were placed in the conventional lead II position. Equipment and settings: ECG was recorded by BioAmp connected to the Powerlab, and data were processed with Chart5 (ADInstruments, CO, U.S.A). We subjected both groups of animals to pharmacologic stress by administering Isoproterenol (ISO) (300 µg of ISO/20 g animal body weight) intra-peritoneally in P28 KI-Cre (n = 8) and WT (n = 8) mice and recorded ECG pre- and post- ISO injection with the protocol described above.

### Statistical Analysis

Data are presented as Mean ± S.E.M. Statistical analysis on the two groups of animals was performed using a unpaired Mann-Whitney non-parametric test, and probability values (p-value) > 0.05 were not considered statistically significant. The statistical calculations were performed using GraphPad Prism 6 software.

### AVCS Single Cell (sc) Preparation for sc-RNA Sequencing

P0 hearts were dissected in ice-cold 1X PBS. tdTomato fluorescence was used to screen KI-Cre animal hearts from the WT hearts. AVCS was microdissected from KI-Cre hearts (n = 3) guided by tdTomato expression under epifluorescent microscope. Microdissected tissue pieces were pooled from (n = 3) KI-Cre hearts. In order to obtain single cell suspension of microdissected AVCS, we used the Pierce Cardiomyocyte Isolation Kit (Thermoscientific, #88281). The cell yield and viability was determined using hemocytometer counting chambers.

### 10X Genomics Library preparation from AVCS SC preparation

10x Genomics’ scRNA-Seq library was constructed following the manufacturer’s instructions (Zheng *et al*., 2017). Briefly, Single cell suspension was washed with 1X PBS (0.04% BSA) before counting. The concentration of single cell suspension was adjusted to about 500 to 1000 cells/µL and was loaded on the 10x Genomics’ Chromium™ system (10x Genomics, Pleasanton, CA) with the aim of generating 6000 to 10000 transcriptomes per channel (Chromium™ Single Cell 3′ Library & Gel Bead Kit v2, catalog number 120237). Illumina NextSeq. 500 was used for paired-end sequencing of the library. Average insert size was about 400 base pairs.

### Bioinformatic Analysis for Single-Cell RNA Sequencing

BCL files generated by Illumina NextSeq500 were demultiplexed and converted to standard FASTQ files using mkfastq function from Cell Ranger pipeline (version 2.1.0) with default parameters. Raw UMI count matrices representing gene expression values were generated using Cell Ranger pipeline (version 2.1.0) with default parameters, except the parameter of expected number of cells, which was adjusted based on individual experiment. Before clustering, low quality cells (with fewer than 200 genes detected) are filtered. Genes detected in fewer than 30 cells or with fewer than 60 UMIs in total across all cells were filtered. The total UMI counts for each cell were median-normalized. The resulting matrix was natural-log-transformed with the addition of a pseudocount of 1 prior taking the log-transformation. The median-normalized natural-log-transformed matrix was centered and scaled per gene. The resulting matrix was used to perform PCA using the prcomp function from built-in R package stats (version 3.5.1). Rtsne package (version 0.13) was used to (non-linearly) embed single cells on a two-dimensional space (initial PCA: disabled, perplexity parameter: 30, iterations: 3000). The input of Rtsne was the selected principal components’ scores calculated above (output of the prcomp function). The Louvain-Jaccard method (Shekhar *et al*., 2016) was used to cluster single cells. The input is the scores of the chosen principal components.

## Supplementary information

Supplementary Information

Video 1

## Data Availability

The single cell RNA sequencing data reported in this paper were deposited in NCBI Gene Expression Omnibus (GEO) under the accession number GSE118932. To review, go to https://www.ncbi.nlm.nih.gov/geo/query/acc.cgi?acc=GSE117795 and enter token gzahweaipbslfqx into the box.
