# Author Correction: Using Gjd3-CreEGFP mice to examine atrioventricular node morphology and composition

**DOI:** 10.1038/s41598-020-60915-5

**Published:** 2020-02-28

**Authors:** Samadrita Bhattacharyya, Jialei Duan, Lin Wang, Boxun Li, Minoti Bhakta, Antonio Fernandez-Perez, Gary C. Hon, Nikhil V. Munshi

**Affiliations:** 1 Department of Internal Medicine, Division of Cardiology, UT Southwestern Medical Center, Dallas, TX 75390 USA; 2 Laboratory of Regulatory Genomics, Cecil H. and Ida Green Center for Reproductive Biology Sciences, Division of Basic Reproductive Biology Research, Department of Obstetrics and Gynecology, University of Texas Southwestern Medical Center, Dallas, TX 75390 USA; 3 Department of Molecular Biology, UT Southwestern Medical Center, Dallas, TX 75390 USA; 4 McDermott Center for Human Growth and Development, Ut Southwestern Medical center, Dallas, TX 75390 USA; 5 Hamon Center for Regenerative Science and Medicine, Dallas, TX 75390 USA

Correction to: *Scientific Reports* 10.1038/s41598-019-38683-8, published online 14 February 2019

This Article contains a typographical error in the Data Availability section where,

“The single cell RNA sequencing data reported in this paper were deposited in NCBI Gene Expression Omnibus (GEO) under the accession number GSE118932. To review, go to https://www.ncbi.nlm.nih.gov/geo/query/acc.cgi?acc=GSE117795 and enter token gzahweaipbslfqx into the box.”

should read:

“The single cell RNA sequencing data reported in this paper were deposited in NCBI Gene Expression Omnibus (GEO) under the accession number GSE118932. To review, go to https://www.ncbi.nlm.nih.gov/geo/query/acc.cgi?acc= GSE118932.”

