# Supplementary material for: Using Gjd3-CreEGFP mice to examine atrioventricular node morphology and composition
Source: Sci Rep. 2019 Feb 14;9:2106. doi: 10.1038/s41598-019-38683-8 (PMC6375990; doi:10.1038/s41598-019-38683-8)
Supplement: Supplementary file 1 — Supplementary Information [file 41598_2019_38683_MOESM1_ESM.pdf]

Using Gjd3-CreEGFP mice to examine atrioventricular node morphology and composition

Samadrita Bhattacharyya[1], Jialei Duan[2], Lin Wang[1], Boxun Li[2], Minoti Bhakta[1], Antonio Fernandez-Perez[1], Gary C. Hon[2,5], and Nikhil V. Munshi[1,3,4,5,*]

SUPPLEMENTARY INFORMATION

Table S1. Summary of recombination efficiency mediated by the Gjd3-CreEGFP allele.

Stage	tdTomato$^+$ (# Cells)	Cx30.2$^+$ (# Cells)	tdTomato/Cx30.2 Overlap (%)
E12.5	5	4	125
P1	245	232	106
P4	177	201	88
P7	225	220	102
P28	252	200	126
P42	273	284	96

Table S2. List of genotyping primers.

Names of primers	Sequences of primers
F1	5' CTTCGCTTCTTAAGGCCTCCATATG 3'
R1	5' TTATTCCAAGCGGCTTCGGC 3'
F2	5' ATATTGCTGAAGAGCTTGGCGGC 3'
R2	5' TGTTAGGGTGGGTGTCCAGCTTG 3'
F3	5' GCTGCTGCCCGACAACCACTA 3'
R3	5' AGGGTACTGTTGGGAGAAGGTGTCT 3'
F4	5' GCATTACCGGTCGATGCAACGAGTGATGAG 3'
R4	5' GAGTGAACGAACCTGGTCGAAATCAGTGCG 3'

SUPPLEMENTARY FIGURE LEGENDS

Figure S1. Confirmation of knock-in cassette insertion and FLP-mediated recombination in Gjd3$^{3'UTR-IRES-CreEGFP/+}$ mice. a) PCR screening before FLP-mediated recombination to demonstrate proper targeting of the knock-in cassette. i) PCR products generated by primer set F1-R1 designed to amplify Neo^R and the beginning of the Gjd3 3'UTR. Positive F1 mice (2, 3, and 6) displayed both the wild-type (632 bp) and recombined (495 bp) allele fragments. ii) The 3 positive PCRs were confirmed with a second primer set (F2-R2) that generates a 546 bp recombined allele fragment. b) Following FLP-mediated recombination and Neo^R cassette deletion, PCR primer set F3-R3 was used to identify a 372 bp band that indicates Neo^R removal in 3 pups (2,3, and 6). c) The presence of the CreEGFP transgene was confirmed in the 3 positive mice using universal Cre primers (F4-R4) that generate a 410 bp product. d) Representative Sanger sequencing results confirming appropriate genomic targeting and fidelity of the 3' KI cassette boundary. Location of genotyping primers are shown in Figure 1.

Figure S2. WT hearts do not display tdTomato fluorescence. Whole mount fluorescent imaging of dissected hearts from E12.5, E16.5, P0, P7, P14, P21, and P28 WT mice exhibit no reporter expression. Number of hearts dissected n= 8 for every developmental time-point. Scale bar: 500 µm.

Figure S3. Establishment of IHC protocol for GFP on frozen mouse heart sections. Using Tyramide Signal Amplification (TSA) method for GFP IHC on P4 Gjd3$^{3'UTR-IRES-CreEGFP/+}$; R26R$^{tdTomato/+}$ heart cryosections, (i) complete overlap of GFP signal (green) and Cre-recombined tdTomato (red) was observed. (ii-iv) Negative control IHC experiments on P4 Gjd3$^{3'UTR-IRES-CreEGFP/+}$; R26R$^{tdTomato/+}$ heart cryosections demonstrating lack of non-specific GFP signal without

(i) Labeled Tyramide A488, (ii) secondary antibody (HRP conjugated), or (iii) primary antibody (anti-GFP) incubations. Scale bar: 100 µm.

Figure S4. Gjd3-CreEGFP mediated recombination overlaps with GFP at P7. GFP IHC on P7 $Gjd3^{3'UTR-IRES-CreEGFP/+}$; $R26R^{tdTomato/+}$ heart cryosections showed nearly complete overlap of GFP signal (green) and Cre-recombined tdTomato (red). Scale bar: 100 µm.

Figure S5. Gjd3-CreEGFP labels embryonic AVC at E12.5. i) Sagittal cryosection of an E12.5 $Gjd3^{3'UTR-IRES-CreEGFP/+}$; $R26R^{tdTomato/+}$ embryo was stained for Cx30.2 (green) to confirm overlap with tdTomato (red). ii) Sister section from the same E12.5 $Gjd3^{3'UTR-IRES-CreEGFP/+}$; $R26R^{tdTomato/+}$ embryo was stained for Tbx3 (green) to demonstrate that tdTomato (red) localizes to the developing AVC (yellow arrow). Dashed yellow line outlines the developing heart, and white arrowheads indicate $tdTomato^{+}$ cells. Nuclei were counterstained with DAPI (blue). A, Atrium; V, Ventricle. Scale bar: 100 µm.

Figure S6. The $Gjd3^{3'UTR-IRES-CreEGFP/+}$ allele mediates infrequent recombination in the right ventricle and outflow tract (OFT) at E16.5. Sister sections from an E16.5 $Gjd3^{3'UTR-IRES-CreEGFP/+}$; $R26R^{tdTomato/+}$ heart following permeabilization and counter-staining with DAPI showed sparse $tdTomato^{+}$ cells (yellow arrows) in the RV and OFT. RV, Right Ventricle; LV, Left Ventricle; LVOT, Left Ventricle Outflow Tract; RVOT, Right Ventricle Outflow Tract. Scale bar: 100 µm.

Figure S7. Cre-mediated recombination in the AVN of $Gjd3^{3'UTR-IRES-CreEGFP/+}$; $R26R^{lacZ/+}$ mice. Freshly dissected P7 whole hearts were fixed and stained with X-gal. Stained hearts were sectioned and counterstained with NFR. Brightfield microscopic images are shown for different sections, demonstrating lacZ expression throughout the AVN. Scale bar: 100 µm.

Figure S8. Preserved Gjd3 protein levels in $Gjd3^{3'UTR-IRES-CreEGFP/+}$ mice. (a) Extracted total protein from whole heart tissue of P42 $Gjd3^{3'UTR-IRES-CreEGFP/+}$ and WT littermates (pooled from n=3 hearts each) was used for Western Blot analysis. The protein samples were derived from the same experiment and all the blots were processed in parallel. Blots were probed with antibodies for Cx30.2 (predicted size 30 kilo Daltons (kD) and Hcn4 (predicted size ~129 kD). α-Tubulin was used as a loading control and ran in the same experiment. b) Blots were quantified by ImageJ and confirmed that any changes in Cx30.2 or Hcn4 expression were not significant (n.s.; p-value=0.343) between the two groups. c) Immunofluorescence images on P42 heart cryosections from $Gjd3^{3'UTR-IRES-CreEGFP/+}$ and WT littermates confirming preservation of endogenous Cx30.2 and Hcn4 expression. Scale bar: 100 µm. Images were cropped using Adobe Photoshop CS6. Full-length blots are presented in Supplementary Figure S9.

Figure S9. Full-length Western blots for Figure S8a.

Figure S10. $Gjd3^{3'UTR-IRES-CreEGFP/+}$ allele mediated tdTomato expression overlaps with endogenous Cx30.2 in the AVN. Sections were obtained from $Gjd3^{3'UTR-IRES-CreEGFP/+}$; $R26R^{tdTomato/+}$ mice at E12.5 (Sagittal section of whole embryo), P1, P4, P7, P28, and P42 for staining with Cx30.2 antibody (green). Cx30.2 staining highly correlates with recombined tdTomato (red) signal across all stages. $Cx30.2^{+}$ AVN regions are outlined in white. Scale bars: 100 µm.

Figure S11. $Gjd3^{3'UTR-IRES-CreEGFP/+}$ hearts maintain structural integrity. Hematoxylin and Eosin (H&E) stained heart sections from P42 wild-type (WT) and $Gjd3^{3'UTR-IRES-CreEGFP/+}$ littermate mice in the four-chamber orientation. Comparison of slides confirms overall structural and morphological integrity in $Gjd3^{3'UTR-IRES-CreEGFP/+}$ mice. Scale bar: 500 µm.

Figure S12. Minimal alteration of atrioventricular conduction in homozygous Gjd3-CreEGFP mice. Representative ECG tracings are shown for a wild-type (a, blue) and a Gjd3-CreEGFP heterozygous (b, gray) mouse. For comparison, ECG tracings for three individual adult Gjd3-CreEGFP homozygous mice (red) are shown (c i-iii). Dashed vertical line shows the onset of the P wave, and the PR interval is indicated by a double-headed arrow. Individual PR interval measurements are shown at the top of each tracing.

Figure S13. Distribution of established AVN markers in the P0 AVC CM atlas. Expression of known AVN markers (Hcn4, Tbx3, and Tbx2) was mapped onto t-SNE plots of AVC CMs. Colored scale: Expression of transcripts in log2 scale; darker indicates low or no expression and lighter color indicates higher expression.

Video 1. Three-dimensional reconstruction of the P0 Cx30.2-tdTomato^{+} AVN. A P0 Cx30.2-tdTomato heart was processed by CUBIC clearing and imaged by confocal microscopy. The IMARIS software package was used to generate a video of the three-dimensional model.

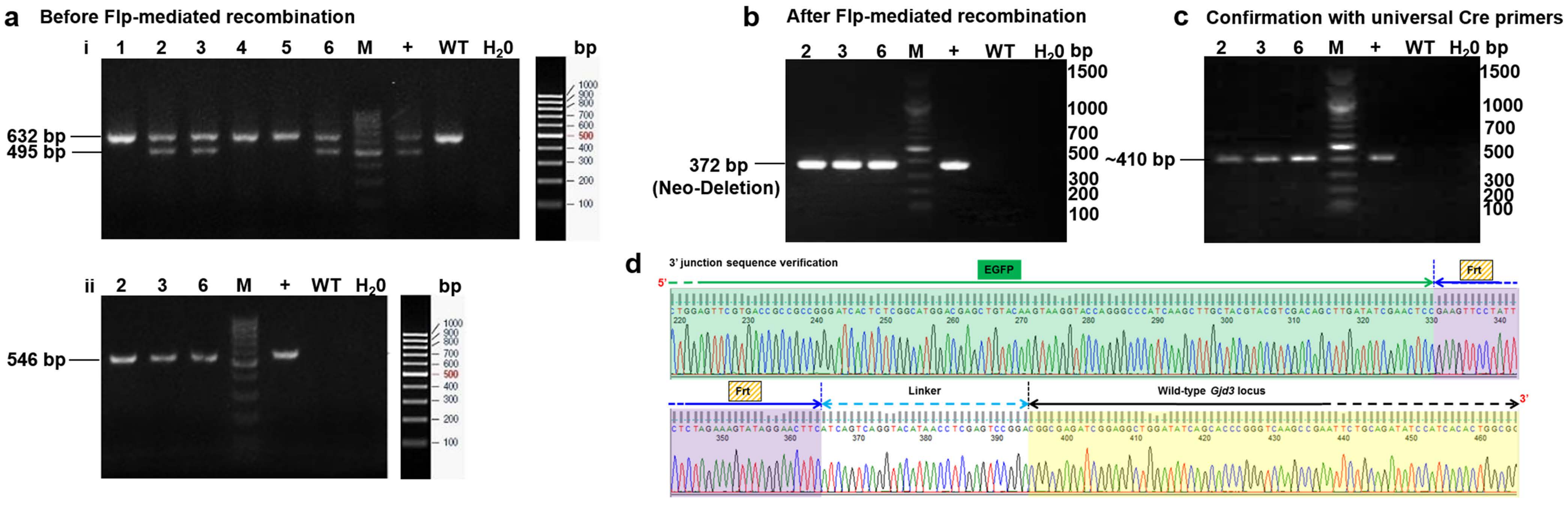
a
Before Flp-mediated recombination
i
1
2
3
4
5
6
M
+
WT
H20
bp
632 bp
495 bp
1000
900
800
700
600
500
400
300
200
100
ii
2
3
6
M
+
WT
H20
bp
546 bp
b
After Flp-mediated recombination
2
3
6
M
+
WT
H20 bp
1500
1000
700
500
300
200
100
372 bp
(Neo-Deletion)
c
Confirmation with universal Cre primers
~410 bp
d
3' junction sequence verification
EGFP
Frt
5'
3'
Linker
Wild-type Gjd3 locus

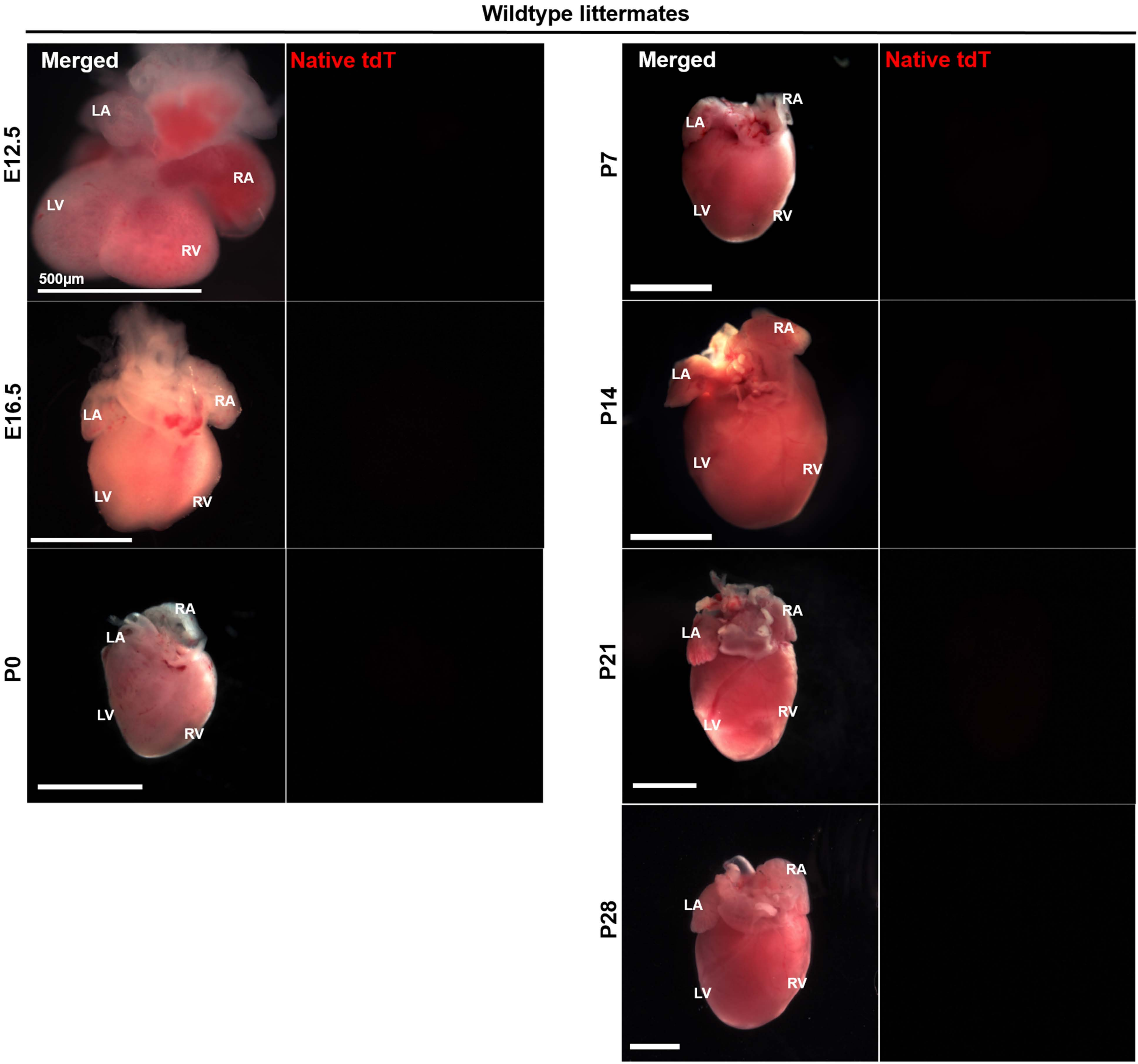
Wildtype littermates
Merged
Native tdT
E12.5
LA
RA
LV
RV
500µm
E16.5
LA
RA
LV
RV
P0
RA
LA
LV
RV
Merged
Native tdT
P7
RA
LA
LV
RV
P14
RA
LA
LV
RV
P21
RA
LA
RV
LV
P28
RA
LA
RV
LV

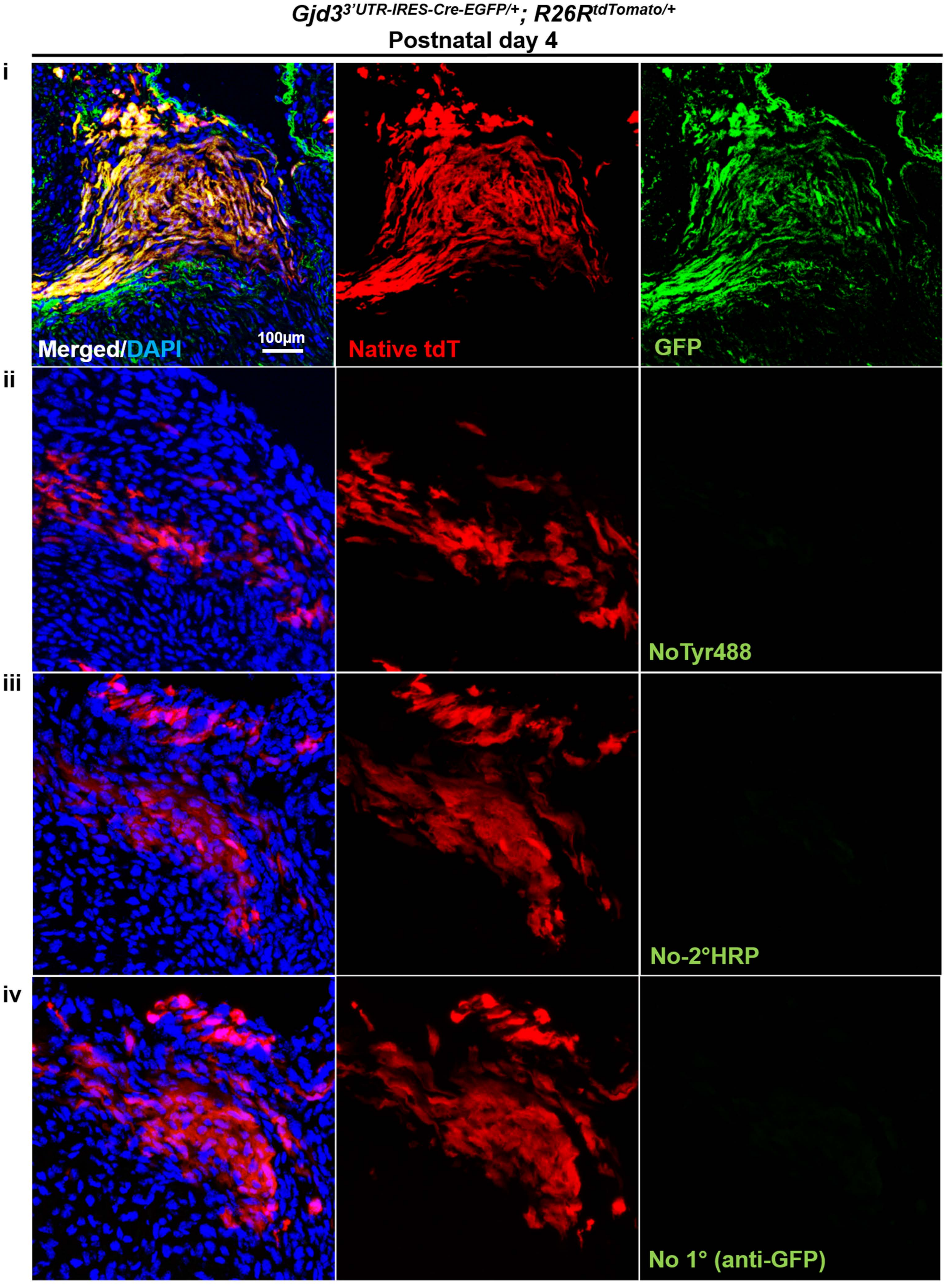

Gjd3 3'UTR-IRES-Cre-EGFP/+; R26R tdTomato/+
Postnatal day 4
i
ii
iii
iv
Merged/DAPI
100μm
Native tdT
GFP
NoTyr488
No-2°HRP
No 1° (anti-GFP)

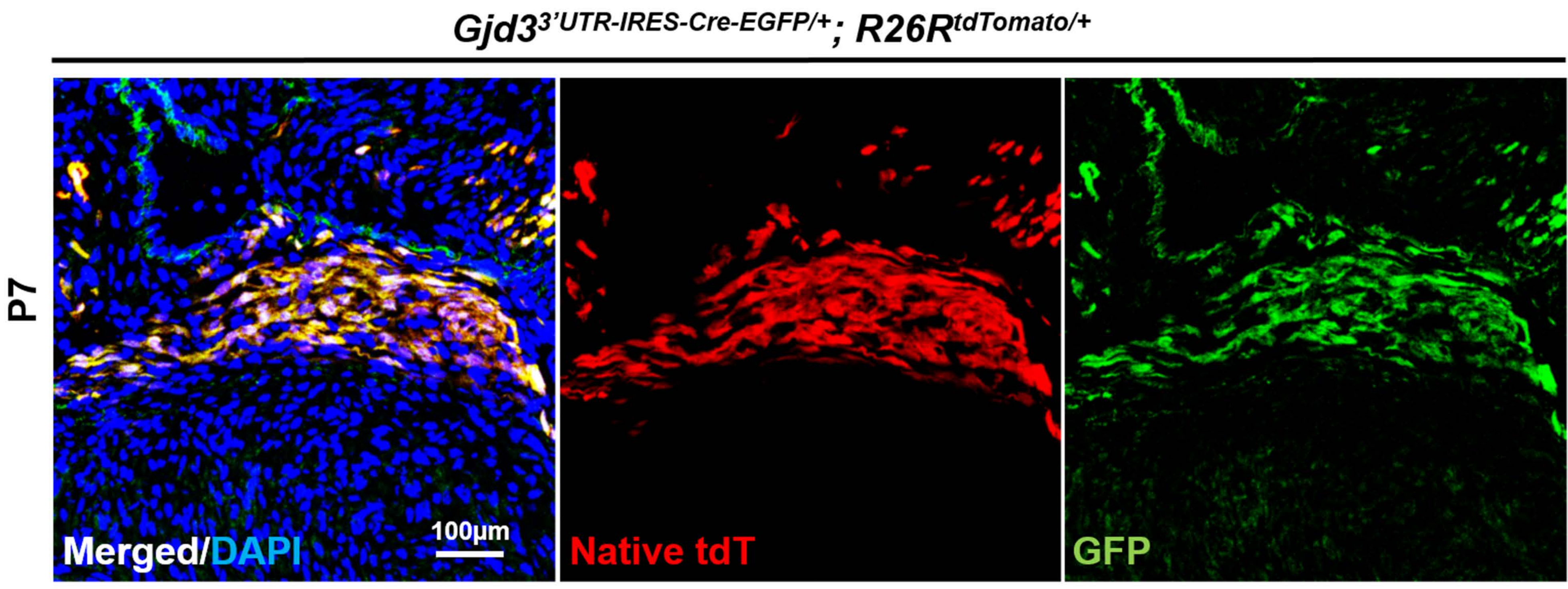

Gjd3 3'UTR-IRES-Cre-EGFP/+; R26R tdTomato/+
P7
100μm
Merged/DAPI
Native tdT
GFP

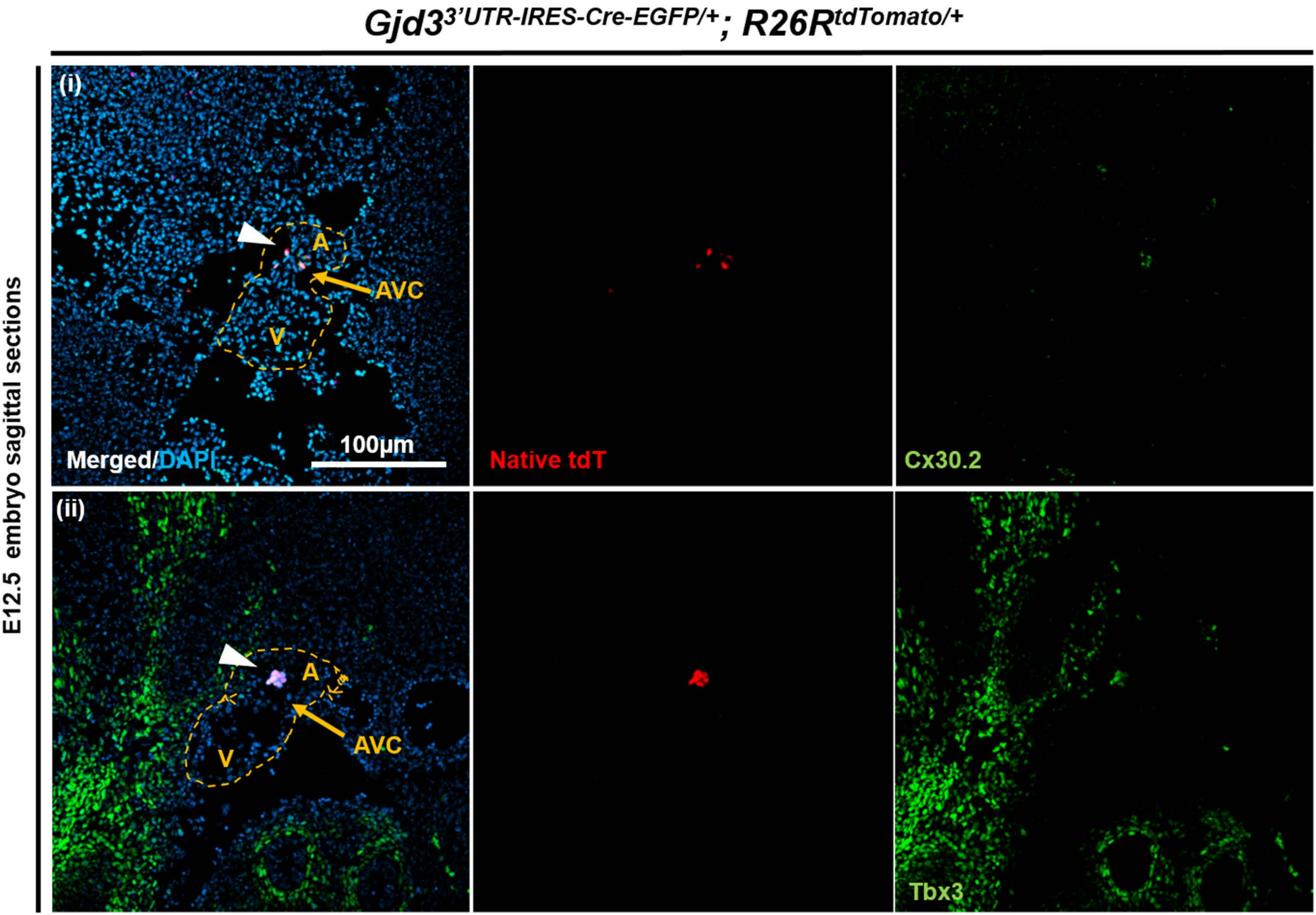

Gjd3^3'UTR-IRES-Cre-EGFP/+; R26R^tdTomato/+
E12.5 embryo sagittal sections
(i)
A
AVC
V
100μm
Merged/DAPI
Native tdT
Cx30.2
(ii)
A
AVC
V
Tbx3

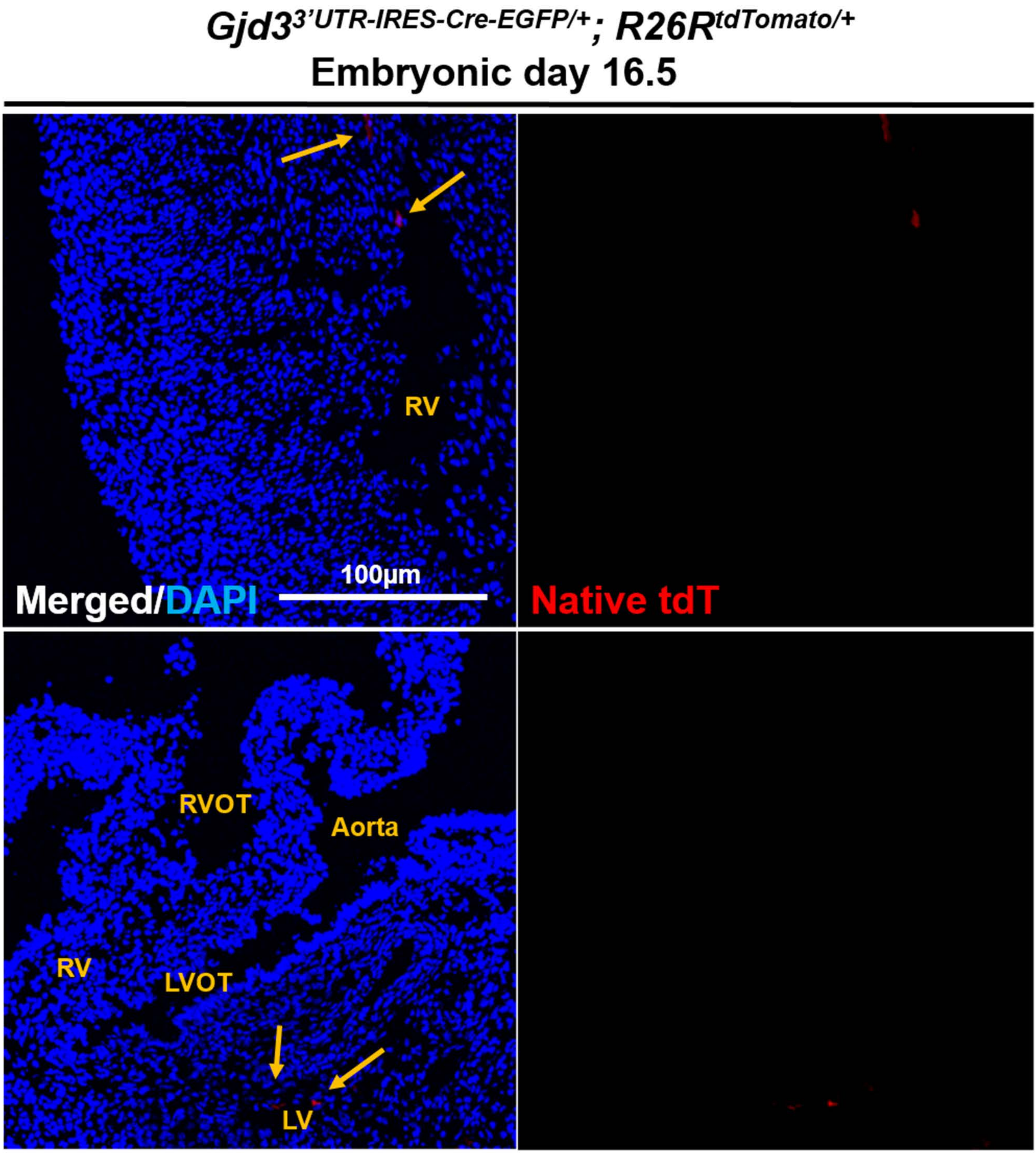
Gjd3 3'UTR-IRES-Cre-EGFP/+; R26R tdTomato/+
Embryonic day 16.5
RV
100μm
Merged/DAPI
Native tdT
RVOT
Aorta
RV
LVOT
LV

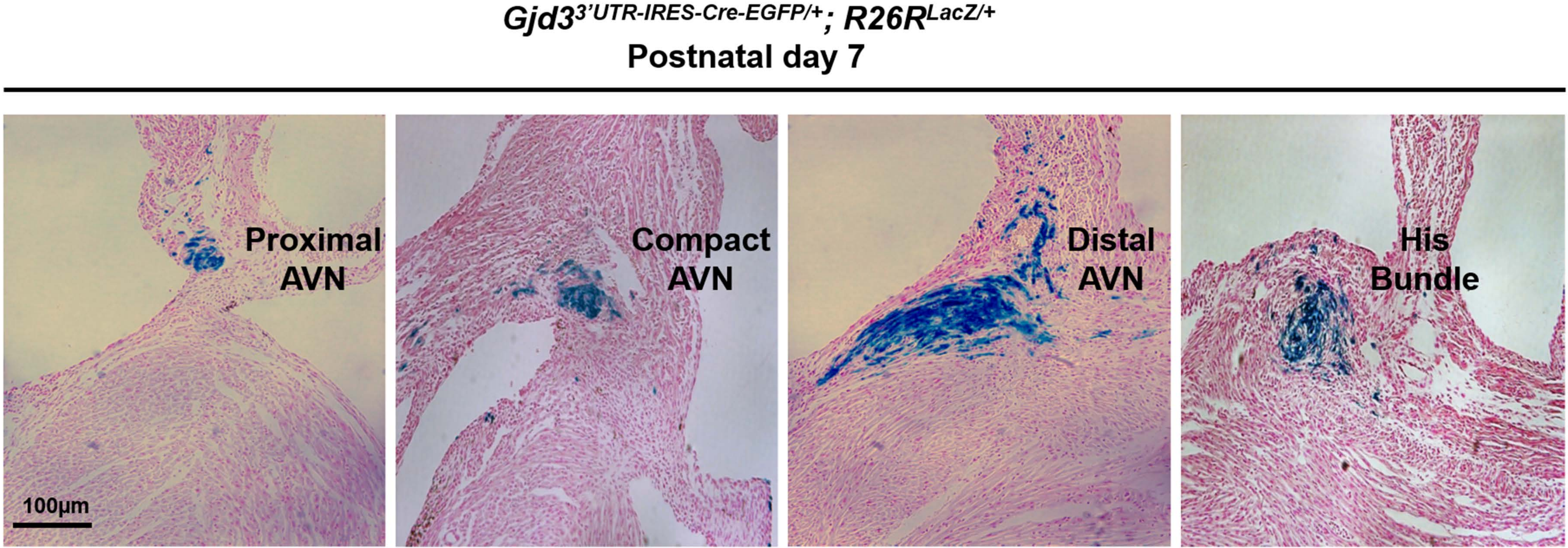
Gjd3 3'UTR-IRES-Cre-EGFP/+; R26R LacZ/+
Postnatal day 7
Proximal AVN
Compact AVN
Distal AVN
His Bundle
100μm

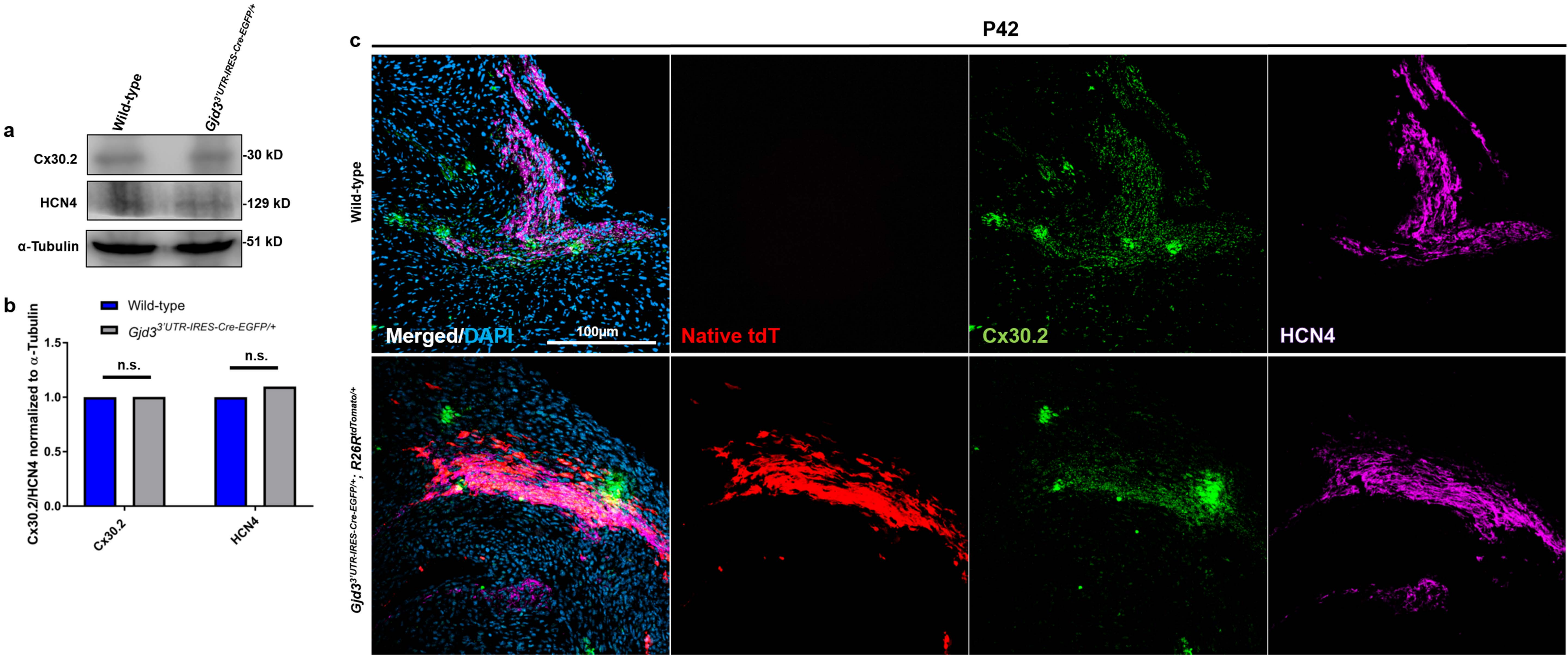
a
Wild-type
Gjd3^3'UTR-IRES-Cre-EGFP/+
Cx30.2
HCN4
α-Tubulin
-30 kD
-129 kD
-51 kD
b
Wild-type
Gjd3^3'UTR-IRES-Cre-EGFP/+
Cx30.2/HCN4 normalized to α-Tubulin
1.5
1.0
0.5
0.0
n.s.
n.s.
Cx30.2
HCN4
c
P42
Wild-type
Gjd3^3'UTR-IRES-Cre-EGFP/+; R26R^tdTomato/+
Merged/DAPI
100μm
Native tdT
Cx30.2
HCN4

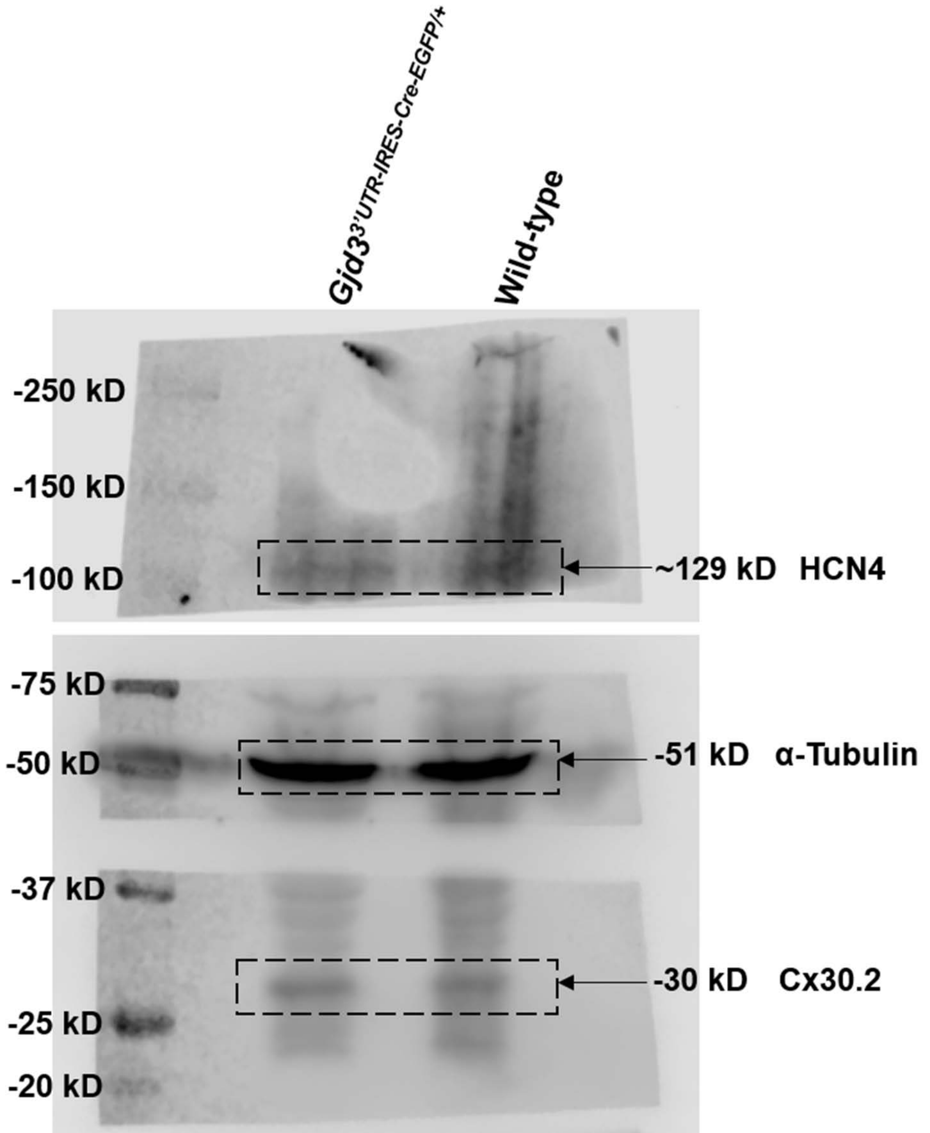

Gjd3 3'UTR-IRES-Cre-EGFP/+
Wild-type
-250 kD
-150 kD
-100 kD
~129 kD HCN4
-75 kD
-50 kD
-51 kD α-Tubulin
-37 kD
-25 kD
-20 kD
-30 kD Cx30.2

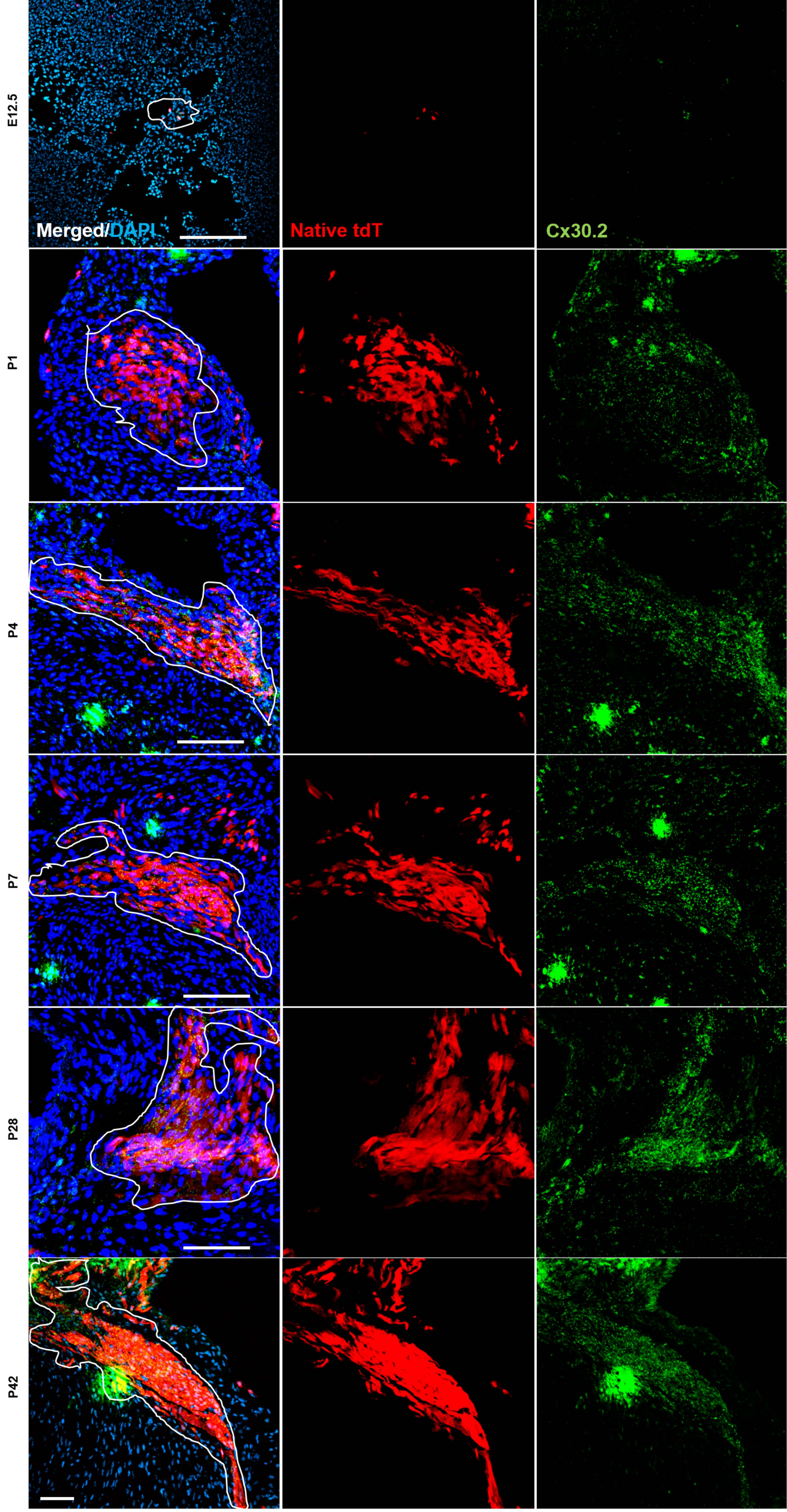
E12.5
Merged/DAPI
Native tdT
Cx30.2
P1
P4
P7
P28
P42

[illegible]

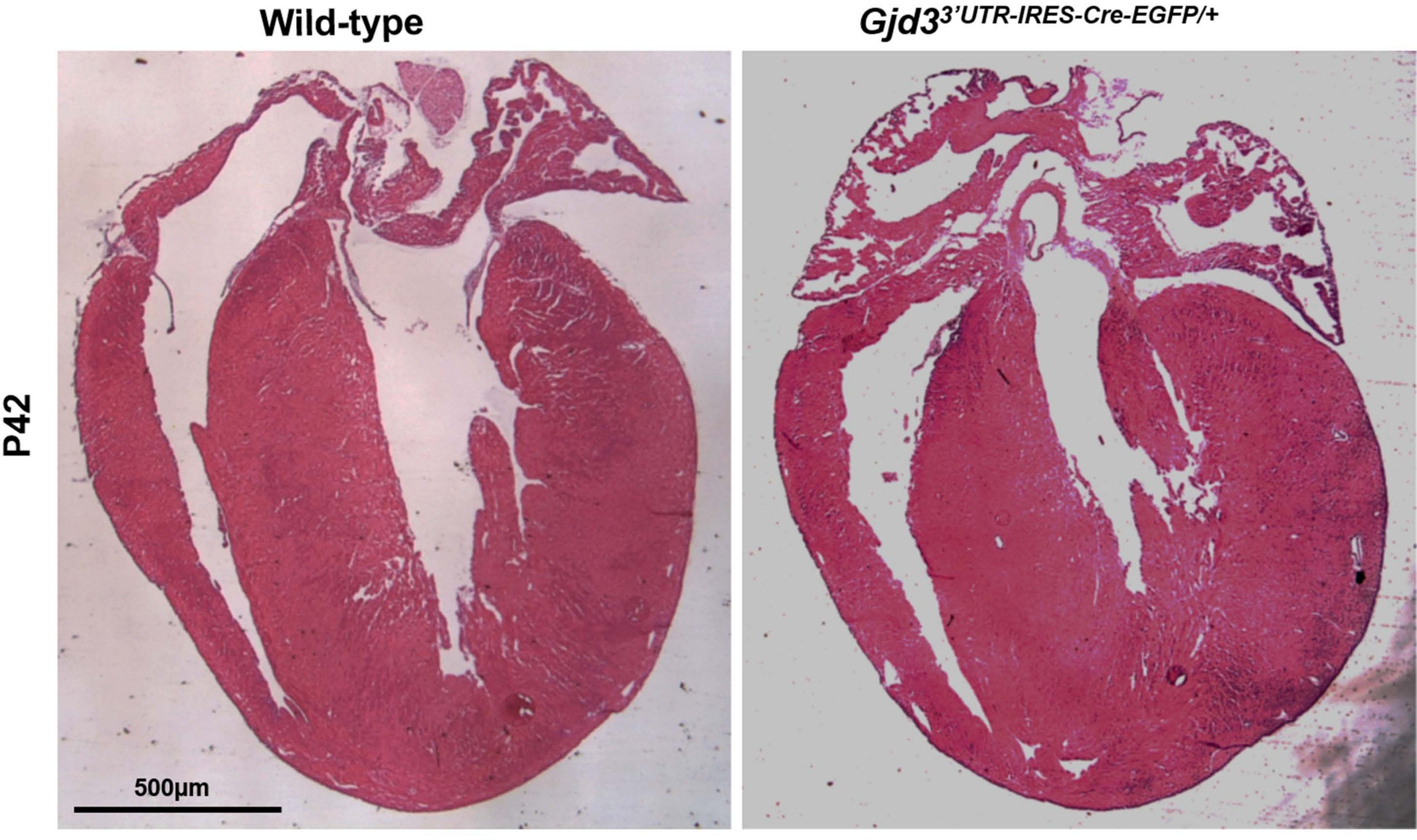
Wild-type
Gjd3$^{3'UTR-IRES-Cre-EGFP/+}$
P42
500µm

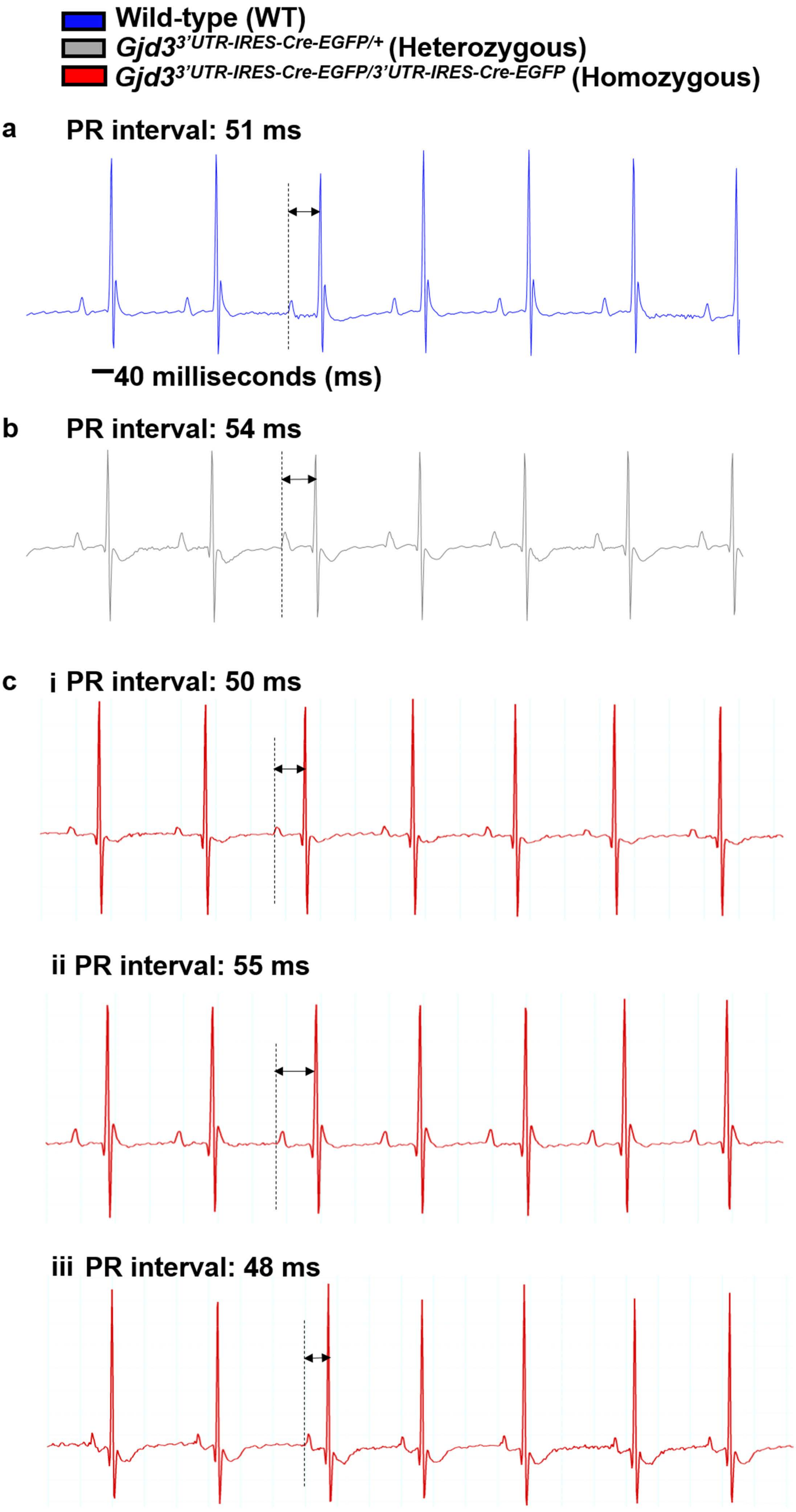

Wild-type (WT)
Gjd3 3'UTR-IRES-Cre-EGFP/+ (Heterozygous)
Gjd3 3'UTR-IRES-Cre-EGFP/3'UTR-IRES-Cre-EGFP (Homozygous)
a
PR interval: 51 ms
40 milliseconds (ms)
b
PR interval: 54 ms
c
i PR interval: 50 ms
ii PR interval: 55 ms
iii PR interval: 48 ms

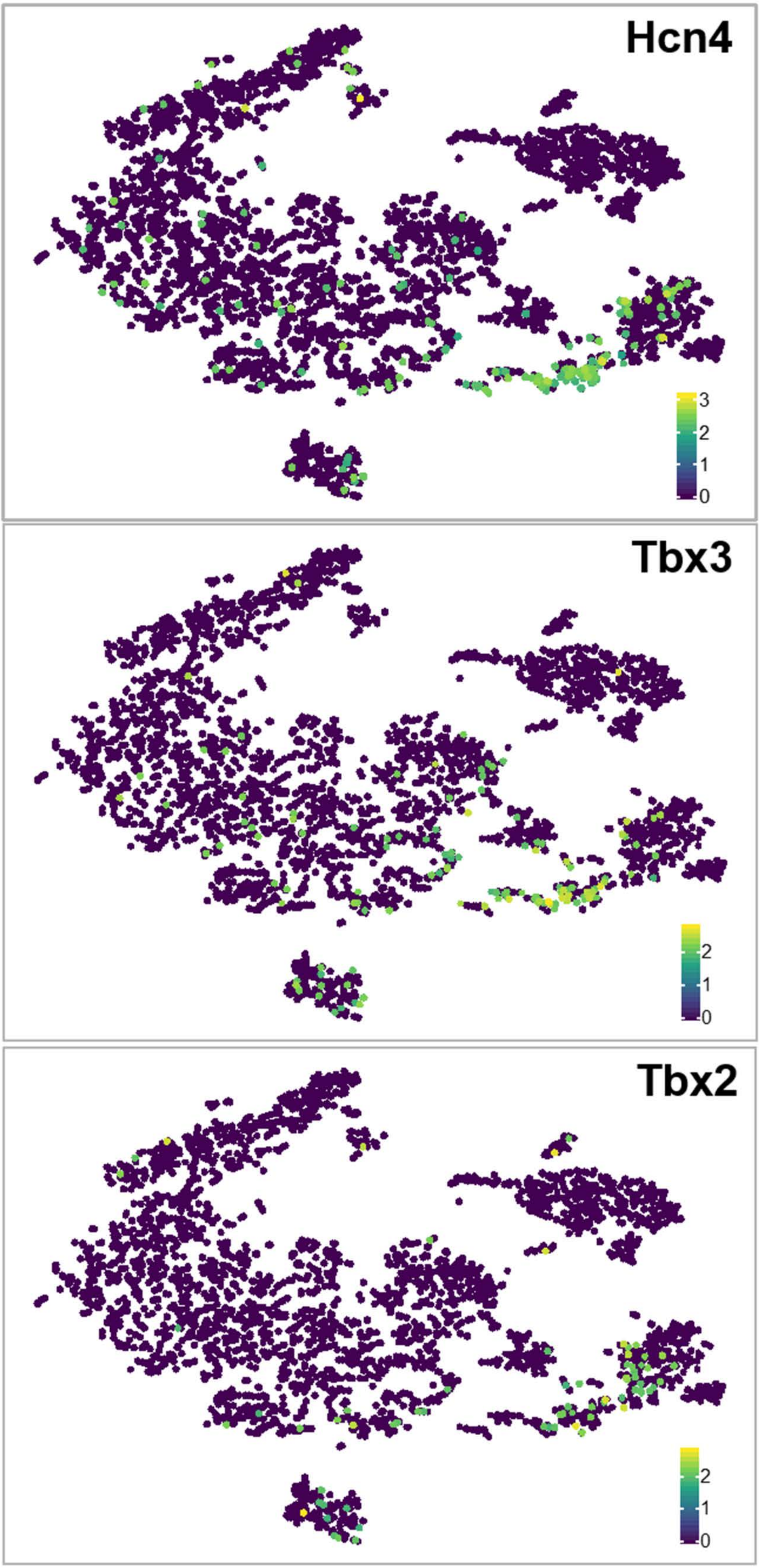
Hcn4
3
2
1
0
Tbx3
2
1
0
Tbx2
2
1
0
